# lncRNA *miat* functions as a ceRNA to upregulate *sirt1* by sponging *miR-22-3p* in HCC cellular senescence

**DOI:** 10.18632/aging.102240

**Published:** 2019-09-10

**Authors:** Lijun Zhao, Kexin Hu, Jianzhong Cao, Pan Wang, Jun Li, Kewu Zeng, Xiaodong He, Peng-Fei Tu, Tanjun Tong, Limin Han

**Affiliations:** 1 Peking University Research Center on Aging, Department of Biochemistry and Molecular Biology, School of Basic Medical Sciences, Peking University Health Science Center, Beijing Key Laboratory of Protein Posttranslational Modifications and Cell Function, Beijing 100191, China; 2 Department of General Surgery, Peking Union Medical College Hospital, Chinese Academy of Medical Sciences, Peking Union Medical College, Beijing 100191, China; 3 Department of Laboratory Medicine, Peking University Third Hospital, Beijing 100191, China; 4 Modern Research Center for Traditional Chinese Medicine, School of Chinese Materia Medica, Beijing University of Chinese Medicine, Beijing 100029, China; 5 Modern Research Center for Traditional Chinese Medicine, Beijing University of Chinese Medicine, Beijing 100029, PR China; 6 State Key Laboratory of Natural and Biomimetic Drugs, School of Pharmaceutical Sciences, Peking University Health Science Center, Beijing 100191, China

**Keywords:** long noncoding RNA miat, ceRNA, hepatocellular carcinoma, cell senescence

## Abstract

Hepatocellular carcinoma (HCC) is a leading cause of cancer related deaths and lacks effective therapies. Cellular senescence acts as a barrier against cancer progression and plays an important role in tumor suppression. Senescence associated long noncoding RNAs (SAL-RNAs) are thought to be critical regulators of cancer development. Here, the long noncoding RNA (lncRNA) myocardial infarction-associated transcript *(miat*) was first identified as an HCC specific SALncRNA. Knockdown of *miat* significantly promoted cellular senescence and inhibited HCC progression. Mechanistic study revealed that SAL-*miat* acted as a competitive endogenous RNA (ceRNA) that upregulated the expression of *sirt1* by sponging *miR-22-3p*. Moreover, *miat* downregulation activated the tumor suppressor pathway (p53/*p21* and *p16*/pRb) and stimulated senescent cancer cells to secrete senescence-associated secretory phenotype (SASP), which contributed to inhibition of tumor cell proliferation, and resulted in the suppression of HCC tumorigenesis. Together, our study provided mechanistic insights into a critical role of *miat* as a miRNA sponge in HCC cellular senescence, which might offer a potential therapeutic strategy for HCC treatment.

## INTRODUCTION

Hepatocellular carcinoma (HCC) represents one of the highest morbidity and mortality occurring cancers worldwide [1, 2]. Despite continued improvement in both improving early detection and developing novel therapeutic strategies, the prognosis of HCC patients remained poor [3]. Therefore, it is vital to dissect the detailed molecular mechanisms regulating the aggressive behaviours of HCC to benefit novel therapeutics targeting HCC.

Aging is a universal phenomenon and can’t be reversed. It is defined as the gradual degeneration of the physical, psychological, and biological state over time, starting after reaching adulthood (reproductive maturity), and eventually leading to disease [4]. Aging is considered as a major risk factor in various diseases, such as neurological disorders, cardiovascular malfunctions, metabolic disruptions, immunological abnormalities and cancer [5]. Cellular senescence is defined as a state of irreversible growth arrest and leads to a senescent phenotype, which is characterized by upregulated senescence-associated β-galactosidase (*SA-β-gal*) activity, cell cycle arrest and cell proliferation inhibition [6, 7]. It is reported that cellular senescence is a key obstacle to the initiation and progression of HCC [8]. Therefore, anticancer treatments based on pro-senescent therapies should be a promising strategy.

Long noncoding RNAs (lncRNAs), >200nt, lack the potential for protein coding [9]. Increasing research has shown that numerous human cancer events associated with dysregulation of lncRNAs, and several lncRNAs have been recognized as prognostic biomarkers with positive therapeutic effects in cancers [10–12]. Emerging evidences have shown that many well-known and novel lncRNAs are associated with initiation and progression of senescence in mammals [13, 14]. Therefore, understanding the correlation between the progression of cellular senescence and tumor suppression and exploring the underlying mechanisms would provide a potential means to investigate novel therapeutic strategies for age-related diseases [15].

With this aim, using bioinformatics analysis, we analyzed differentially expressed lncRNAs during replicative senescence and HCC tumorigenesis and focused on the lncRNA myocardial infarction-associated transcript (*miat*). Existing evidences have shown that abnormal expression of LncRNA *miat* is linked to various cancers, such as breast cancer [16], gastric cancer [17], non-small cell lung cancer [18] and neuroendocrine prostate cancer [19]. Study also showed that *miat* silencing led to proliferation defects and senescence phenotype in human fibroblast WI-38 cells [20]. In addition, it is reported that *miat* has a negative regulatory effect on G1/S phase arrest of human cell cycle, suggesting that *miat* may play an important role in the regulation of cell proliferation. Here, we found that the expression level of *miat* decreased in cellular senescence, and that *miat* silencing significantly promoted cellular senescence. Furthermore, *miat* was frequently upregulated in human HCC and knockdown of *miat* inhibited HCC progression.

LncRNAs can also act as microRNA (miRNA or miR) ‘sponges’, reducing the abundance of their target miRNAs, indirectly regulating gene or mRNA function. MiRNAs are small non-coding RNAs which regulate the expression of target genes at post-transcriptional levels. Currently, studies have shown that *miat* can interact with different miRNAs in a variety of cancers, including *miR-93* [21], *miR-181b* [22], *miR-155-5p* [23] and *miR-124* [24]. According to the prediction of target prediction programs and experimental analysis, we found that *miat* was a potential target of *miR-22-3p* and negatively regulated the expression of *miR-22-3p*. In addition, *miR-22-3p* is essential for sustaining senescence-like phenotypes and inhibiting hepatic induction by the senescence-associated lncRNA *miat* (SAL- *miat*).

Sirtuins (*Sir2*) are NAD+-dependent histone deacetylases (HDAC) in *Saccharomyces cerevisiae*. A number of studies have shown that *sirt1* can delay cellular senescence by inhibiting apoptosis, regulating metabolism (calorie consumption, fat storage, etc.), maintaining normal mitochondrial functions under oxidative stress and inhibiting inflammation [25]. Increasing research suggested that *sirt1* could be a promising therapeutic target for cancer prevention and therapy [26]. In our study, *sirt1* is identified as a direct target of *miR-22-3p*. LncRNA *miat* acted as a competitive endogenous RNA (ceRNA) for *miR-22-3p* to regulate *sirt1* expression. The restoration of *sirt1* expression reversed the cellular senescence and HCC progression induced by *miR-22-3p* and *miat* silencing.

Either or both of the *p53*/*p21* and *p16*/*pRb* tumor suppressive pathways, respond to somewhat different stimuli that induce cellular senescence establish and/or maintain the senescence growth arrest [27–29]. There are multiple upstream regulators, downstream effectors and modified side branches in both pathways, and they also regulate several other features of senescent cells, such as SASP and cell proliferation. Our study found that *miat* silencing inhibited the cell proliferation of HCC cells and stimulated senescent HCC cells to secrete SASP by activating the *p53*/*p21* and *p16*/pRb tumor suppressor pathways.

In summary, our study demonstrated a novel HCC specific SA-LncRNA *miat*, and found that *miat* functions as a ceRNA for *miR-22-3p* to upregulate *sirt1* in HCC cellular senescence. Furthermore, miat downregulation promoted the progression of senescence and activated the tumor suppressor pathway *p53/p21* and *p16/pRb*, which promoted the production of SASP and contributed to tumor cell proliferation inhibition, resulting in inhibition of HCC tumorigenesis.

## RESULTS

### *Miat* was identified as an HCC specific senescence-associated lncRNA

**Figure 1:**
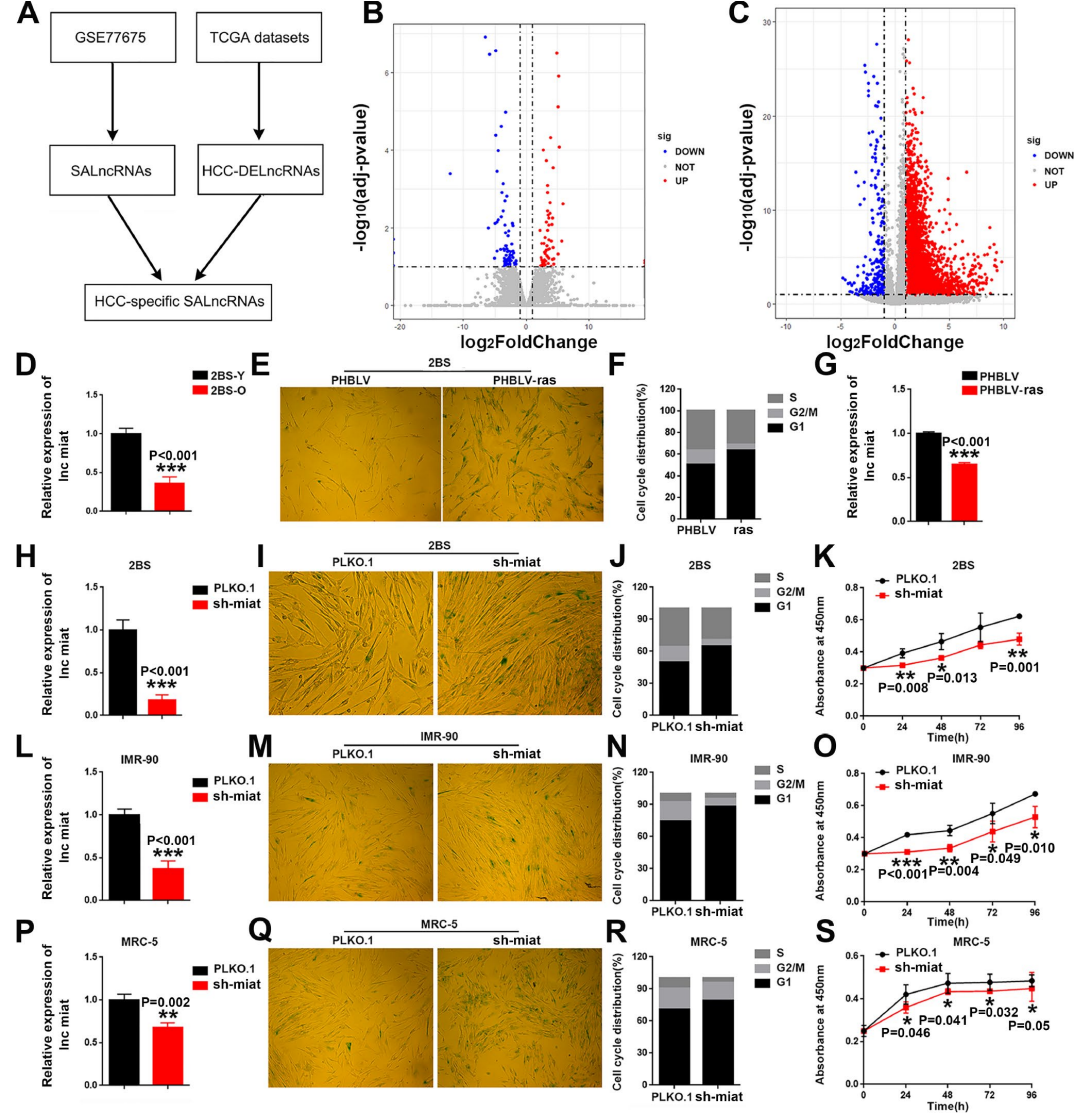
**HCC specific SA-LncRNAs was downregulated during cellular senescence, and *miat* downregulation promoted cellular senescence.** (**A**) Schematic overview of the study design. (**B**, **C**) Volcano plot of differentially expressed genes in proliferating vs. senescent WI-38 cells and HCC vs. normal tissues, respectively. The x-axis indicates log_2_ fold changes between the two groups and the y-axis indicates the -log_10_ adjusted p-value of gene expression variation. The upregulated genes are shown as red dots, the downregulated genes are shown as blue dots and the normal genes are shown as grey dots. (**D**) Real-time PCR analysis for *miat* expression in 2BS cells. The bars represent the mean and SD of three independent experiments, *P < 0.05, **P< 0.01, *** P< 0.001. (**E**) Cellular senescence assay by *SA-β-gal* staining in 2BS cells induced by the oncogene *ras.* (**F**) Cell cycle distribution analysis measured by propidium iodide staining and flow cytometry in 2BS cells induced by the oncogene *ras.* (**G**) Real-time PCR analysis for *miat* expression in 2BS cells induced by the oncogene *ras.* The bars represent the mean and SD of three independent experiments, *P < 0.05, **P< 0.01, *** P< 0.001. (**H**, **L**, **P**) Real-time PCR analysis for *miat* expression in 2BS cells, IMR-90 and MRC-5 cells transfected with the sh-*miat* plasmid. The bars represent the mean and SD of three independent experiments, *P < 0.05, **P< 0.01, *** P< 0.001. (**I**, **M**, **Q**) Cellular senescence assay by *SA-β-gal* staining in 2BS, IMR-90 and MRC-5 cells. (**J**, **N**, **R**) Cell cycle distribution analysis measured by propidium iodide staining and flow cytometry in 2BS, IMR-90 and MRC-5 cells (n=3). (**K**, **O**, **S**) Cell proliferation analysis determined by CCK assay (n=4, mean ± SD) in 2BS, IMR-90 and MRC-5 sh-*miat* cells (n =3, mean ± SD; absorption at 450 nm was detected at 0, 24, 48, 72 h and 96 h after transfection). *P < 0.05, **P< 0.01, *** P< 0.001.

To assess the important role of SALncRNAs in HCC, we used publicly available datasets to analyze DE-lncRNAs during replicative senescence and HCC tumorigenesis (Figure 1A), identifying 111 SALncRNAs (Figure 1B) and 1,997 HCC-DE-lncRNAs (Figure 1C). Then we focused on the HCC-specific SALncRNAs by intersecting the SALncRNAs and HCC-DE-lncRNAs. With the strict screening criteria, only two lncRNAs, namely, *miat* and *cdkn2b-as1*, were identified as HCC-specific SALncRNAs. Compared with *cdkn2b-as1*, *miat* was studied less in both cellular senescence and HCC tumorigenesis. Thus, we focused on the functional importance and detailed mechanisms of *miat* in cellular senescence and HCC tumorigenesis.

### Knockdown of *miat* significantly promoted cellular senescence

To test and verify the potential role of *miat* in cellular senescence, we first measured the expression of *miat* in normal human fibroblast 2BS cells (Figure 1D) and oncogene-induced 2BS senescence (OIS) cell models (Figure 1E–1G). The results showed that, *miat* was downregulated during 2BS cellular senescence. Given that *miat* showed a higher expression level in young 2BS cells, we stably block the expression of *miat* in young 2BS cells using sh RNA-encoding lentiviruses (Figure 1H). As can be seen from the results, compared to the corresponding controls, knockdown of *miat* in 2BS displayed increased perinuclear activity of *SA-β-gal* (Figure 1I), cell cycle arrest (Figure 1J) and cell proliferation inhibition (Figure 1K). Similar results were also acquired in human fibroblast cell lines IMR-90 (Figure 1L–1O) and MRC-5 (Figure 1P–1S). All of these results imply that downregulation of *miat* expression is significant for the induction of senescent phenotypes. Furthermore, to verify that *miat* is an HCC-specific SALncRNA, we generated HCC senescence models by oxidative stress (H_2_O_2_) and DNA damage (doxorubicin, DOX) and explored the role of *miat* in HCC cellular senescence. *SA-β-gal* staining was performed to confirm the establishment of the senescence model induced by different concentrations of H_2_O_2_ and DOX (The data was not shown). We observed a significant decrease of *miat* expression in both HepG2 (Supplementary Figure 1A, 1B) and SMMC-7721 (Supplementary Figure 1C, 1D) senescent models in a manner analogous to dose dependence. These results revealed that *miat* was frequently downregulated during cellular senescence, and *miat* silencing significantly promoted cellular senescence.

### *Miat* was frequently upregulated in human HCC, and *miat* silencing inhibited HCC progression

**Figure 2:**
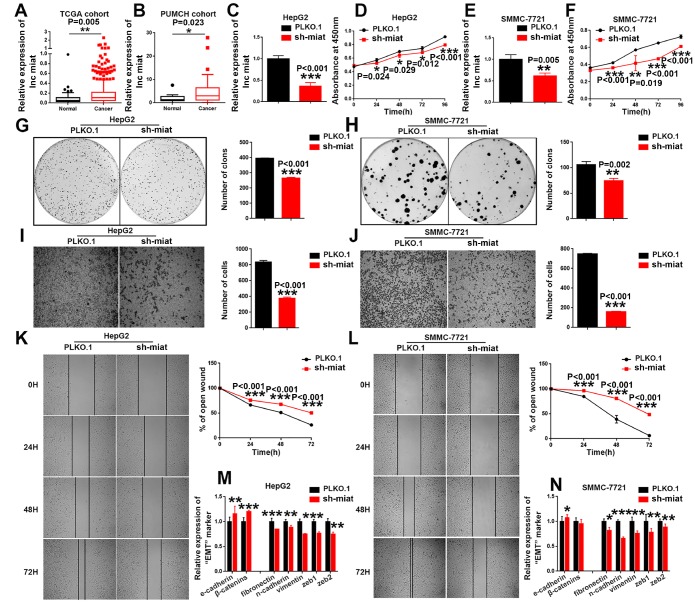
**Knockdown of *miat* suppresses HCC cell hepato-carcinogenesis.** (**A**) LncRNA *miat* expression analyses in HCC and nontumor tissues in TCGA datasets. *P < 0.05, **P< 0.01, *** P< 0.001. (**B**) LncRNA *miat* levels in 20 HCC and paired nontumor tissues. *P < 0.05, **P< 0.01, *** P< 0.001. (**C**, **E**) The mRNA levels of *miat* in *miat*-silenced HepG2 and SMMC-7721 cells. The bars represent the mean and SD of three independent experiments, *P < 0.05, **P< 0.01, *** P< 0.001. (**D**, **F**) Cell proliferation was measured using CCK-8 assays in HepG2 and SMMC-7721 cells with a stable knockdown of *miat*. The bars represent the mean and SD of three independent experiments, *P < 0.05, **P< 0.01, *** P< 0.001. (**G**, **H**) Cell colony formation assay was performed 14 days after stably knockdown *miat* in HepG2 and SMMC-7721 cells, and the colony number per field was calculated (right). The bars represent the mean and SD of three independent experiments, *P < 0.05, **P< 0.01, *** P< 0.001. (**I**, **J**) Transwell assay showing that knockdown of *miat* reduced the migration of HepG2 and SMMC-7721 cells. Representative images of the migratory cells were captured 28 h after the cells were inoculated; *P < 0.05, **P< 0.01, *** P< 0.001. (**K**, **L**) The wound-healing assay demonstrated that *miat* silencing reduced the migration of HepG2 and SMMC-7721 cells. Representative images were captured at 0 h, 24 h, 48 h and 72 h after scratching. The wound closure distance was measured with the software from the Leica Application Suite. The bars represent the mean and SD of three independent experiments, *P < 0.05, **P< 0.01, *** P< 0.001. (**M**, **N**) Real-time PCR analysis of the mRNA levels of key EMT markers was performed in HepG2 and SMMC-7721 cells with a stable knockdown of *miat*. The bars represent the mean and SD of three independent experiments, *P < 0.05, **P< 0.01, *** P< 0.001.

We investigated the functional importance of *miat* in HCC. We first investigated the expression levels of *miat* in HCC and adjacent non-tumor tissues using transcriptome data downloaded from TCGA. Results show that *miat* expression was significantly higher in HCC tissues than in the matched noncancerous hepatic tissues (Figure 2A). We also analysed *miat* expression in a PUMCH cohort containing 20 pairs of HCC and para-tumor tissues. Consistently, the expression of *miat* in the HCC tissue samples was markedly higher than in para-tumor tissues (Figure 2B). In summary, we get the conclusions that *miat* was frequently upregulated in human HCC.

To investigate whether downregulated *miat* expression helps inhibit HCC progression, we stably silenced *miat* in HepG2, SMMC-7721 and Huh7 cells (Figure 2C, 2E and Supplementary Figure 1M). We detected the cell proliferation by CCK8 assays at different time points and found that knockdown of *miat* inhibited HCC cell proliferation (Figure 2D, 2F and Supplementary Figure 1N). These results were further supported by the colony formation assays, which indicated that *miat* knockdown significantly inhibited the HCC cells colony formation (Figure 2G, 2H and Supplementary Figure 1Q, 1R). It has been reported that *miat* was associated with metastasis of solid tumors [30]. Therefore, we conducted the migration ability of *miat* by Transwell and Wound-healing assays. We found that the migration ability of HCC cells was inhibited in sh-miat group (Figure 2I–2L and Supplementary Figure 1O, 1P). Studies also have shown that epithelial-mesenchymal transition (EMT) is involved in multiple biochemical changes of tumor progression [31, 32]. Thus, we detected the mRNA levels of key EMT markers by RT-PCR in *miat* silenced HCC cell lines. We found that typical molecular markers of epithelial cells, *e-cadherin* and *β-catenin*, were up-regulated, whereas the expression of mesenchymal markers, such as *fibronectin*, *n-cadherin*, v*imentin*, *zeb1* and *zeb2* were downregulated in HepG2, SMMC-7721 and Huh7 cells with *miat* knockdown (Figure 2M, 2N and Supplementary Figure 1S). In summary, these results demonstrated that *miat* plays a pro-tumorigenic role in HCC progression.

**Figure 3:**
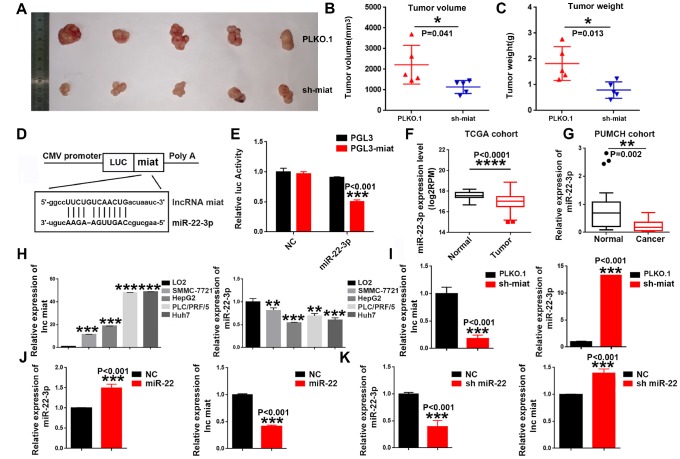
**Knockdown of *miat* suppresses hepatocarcinogenesis in vivo, m*iR-22-3p* bonded to and suppressed *miat* expression.** (**A**–**C**) Subcutaneous injection of SMMC-7721 cells with or without *miat* knockdown into nude mice. Representative images of the resected subcutaneous tumors from each group are shown. Tumor volumes and tumor weights were measured (n = 6). *P < 0.05, **P< 0.01, *** P< 0.001. (**D**) Bioinformatics prediction using miRcode indicated that the *miat* sequence contained the putative binding site of *miR-22-3p*. (**E**) The cDNA of *miat* was cloned downstream of the luciferase gene (PGL3-*miat*) and transfected into HepG2 cells with *miR-22-3p* or control oligonucleotides. Luciferase activity was detected 48 h after transfection. The bars represent the mean and SD of three independent experiments, *P < 0.05, **P< 0.01, *** P< 0.001. (**F**) *MiR-22-3p* expression analyses in HCC and nontumor tissues in TCGA datasets. *P < 0.05, **P< 0.01, *** P< 0.001, **** P< 0.0001. (**G**) *MiR-22-3p* levels in 20 HCC and paired nontumor tissues in PUMCH cohort. *P < 0.05, **P< 0.01, *** P< 0.001, **** P< 0.0001. (**H**) QRT-PCR analysis of *miat* and *miR-22-3p* expression in human normal liver cell line (LO2) and HCC cell lines (SMMC-7721, HepG2, PLC/PRF/5 and Huh7). Data are expressed as the mean ± SD; n=3, *P < 0.05, **P< 0.01, *** P< 0.001, **** P< 0.0001 compared with the control group. (**I**) *MiR-22-3p* expression was increased in HepG2 cells transfected with sh-*miat*. The bars represent the mean and SD of three independent experiments, *P < 0.05, **P< 0.01, *** P< 0.001. (**J**) *Miat* expression was decreased in HepG2 cells transfected with the *miR-22-3p*(miR-22). The bars represent the mean and SD of three independent experiments, *P < 0.05, **P< 0.01, *** P< 0.001. (**K**) *Miat* expression was increased in HepG2 cells transfected with the *miR-22-3p* inhibitor(sh-miR-22); The bars represent the mean and SD of three independent experiments, *P < 0.05, **P< 0.01, *** P< 0.001.

Based on the above data *in vitro,* we hypothesized and verified that the pro-tumorigenesis effect of *miat* on HCC *in vivo*. SMMC-7721 cells with stable sh-*miat* or control cells were collected and subcutaneously injected into nude mice. Tumor size was monitored weekly to observe tumor growth *in vivo.* Consistent with our assumptions, the mice injected with *miat*-silenced HCC cells showed strikingly inhibited tumorigenesis compared with the mice injected with control cells. Representative experimental data of each group are shown in Figure 3A–3C. In conclusion, these data suggested *miat* promoted tumorigenesis of HCC both *in vivo* and *in vitro*.

### *MiR-22-3p* bound to and suppressed *miat* expression

In order to further study the molecular mechanisms of *miat* in HCC, we exploited target prediction algorithms (LncBase Predicted V2.0 and miRWalk 3.0) to predict interaction of LncRNA *miat* and miRNAs. Consistent with existing research results [33], we found that *miat* contains a potential binding site for *miR-22-3p* (Figure 3D). To determine the expression correlation between the two ncRNAs in HCC biology, luciferase reporter assays were conducted. The results of the dual-luciferase reporter assay showed that overexpression of *miR-22-3p* could significantly decrease the luciferase activity of PGL3-*miat* in HepG2 cells (Figure 3E). To confirm the relationship between *miat* and *miR-22-3p*, we detected the *miR-22-3p* expression in HCC tissues and cell lines. Given that *miat* levels were higher in HCC tissues, we observed the down-regulated expression of *miR-22-3p* in HCC tissues from both TCGA database (Figure 3F) and PUMCH database (Figure 3G) when compared with their counterparts. We also measured the expression of *miat* and *miR-22-3p* in a panel of HCC cell lines as wells as normal liver cell line LO2. Similarly, low *miat* and high *miR-22-3p* expression were observed in the normal liver cell line (LO2), while *miat* upregulated and *miR-22-3p* downregulated in most HCC cell lines (Figure 3H). In addition, we found that the expression of *miR-22-3p* significantly increased in sh-*miat* HepG2 cells (Figure 3I). Furthermore, we found that *miat* expression was significantly decreased with *miR-22-3p* overexpression (Figure 3J), and increased after *miR-22-3p* inhibition in HepG2 cells (Figure 3K). These data confirmed the interaction between *miat* and *miR-22-3p* in HCC cell lines, and implied that *miR-22-3p* bound to and suppressed the expression of *miat*.

### *MiR-22-3p* is essential for sustaining senescence-like and tumor-suppressing phenotypes induced by *miat* downregulation

Numerous studies have suggested that some miRNAs inhibit tumor proliferation and promote cellular senescence or ageing. Recently, *miR-22-3p* was considered to be a novel SA-miRNA [34], and a tumor-suppressing miRNA for many types of human cancers [35]. Therefore, we first explored the importance of *miR-22-3p* in cellular senescence and HCC tumorigenesis. The result showed that enforced *miR-22-3p* expression promoted cellular senescence in three human fibroblast cell lines, 2BS, IMR-90 and MRC-5, while down-regulation of *miR-22-3p* impeded the progression of senescence and ameliorated the senescence-like phenotypes (Supplementary Figure 2). We also performed a gain-or-loss of function analysis to explore the role of *miR-22-3p* in the HCC process. Overexpression of *miR-22-3p* significantly inhibited HepG2 cell proliferation, colony formation, migration and EMT *in vitro*, while *miR-22-3p* downregulation had the opposite effect (Supplementary Figure 3). The similar effect was also found in SMMC-7721(Supplementary Figure 4).

**Figure 4:**
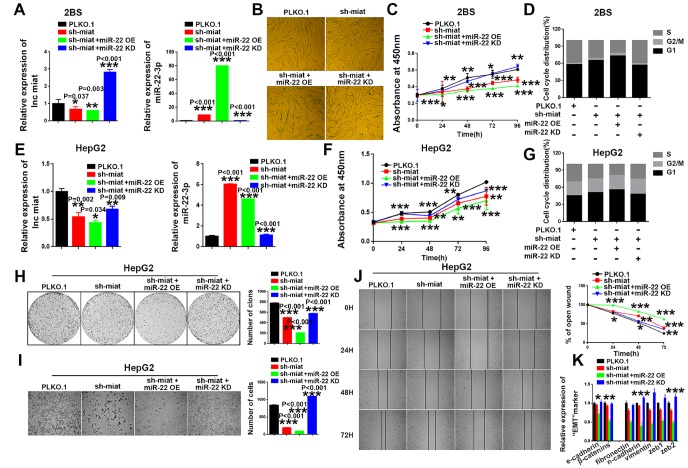
***MiR-22-3p* is essential for sustaining senescence-like phenotypes and inhibiting hepatic induction by sh-*miat*.** (**A**) The mRNA levels of *miat* and *miR-22-3p* in 2BS cells infected with PLKO.1, sh-*miat*, or coinfected sh-*miat* with the *miR-22-3p* (miR-22 OE)/*miR-22-3p* inhibitor (miR-22 KD). The bars represent the mean and SD of three independent experiments, *P < 0.05, **P< 0.01, *** P< 0.001. (**B**) Cellular senescence assay by *SA-β-gal* staining in 2BS cells infected with PLKO.1, sh-*miat*, or coinfected sh-*miat* with the *miR-22-3p* (miR-22 OE)/*miR-22-3p* inhibitor (miR-22 KD). (**C**) Cell proliferation was measured using CCK-8 assays in 2BS cells infected with PLKO.1, sh-*miat*, or coinfected sh-*miat* with the *miR-22-3p* (miR-22 OE)/*miR-22-3p* inhibitor (miR-22 KD). The bars represent the mean and SD of three independent experiments, *P < 0.05, **P< 0.01, *** P< 0.001. (**D**) Cell cycle assay was performed in 2BS cells (n=3) infected with PLKO.1, sh-*miat*, or coinfected sh-*miat* with the *miR-22-3p* (miR-22 OE)/*miR-22-3p* inhibitor (miR-22 KD). (**E**) The expression of *miat* and *miR-22-3p* in HepG2 cells infected with PLKO.1, sh-*miat*, or coinfected sh-*miat* with the *miR-22-3p* (miR-22 OE)/*miR-22-3p* inhibitor (miR-22 KD). The bars represent the mean and SD of three independent experiments, *P < 0.05, **P< 0.01, *** P< 0.001. (**F**) Cell proliferation was measured using CCK-8 assays in HepG2 cells infected with PLKO.1, sh-*miat*, or coinfected sh-*miat* with the *miR-22-3p* (miR-22 OE)/*miR-22-3p* inhibitor (miR-22 KD). The bars represent the mean and SD of three independent experiments, *P < 0.05, **P< 0.01, *** P< 0.001. (**G**) Cell cycle assay was performed in HepG2 cells (n=3) infected with PLKO.1, sh-*miat*, or coinfected sh-*miat* with the *miR-22-3p* (miR-22 OE)/*miR-22-3p* inhibitor (miR-22 KD). (**H**) Cell colony formation assay shown at 14 days after infected with PLKO.1, sh-*miat*, or coinfected sh-*miat* with the *miR-22-3p* (miR-22 OE)/*miR-22-3p* inhibitor (miR-22 KD) in HepG2 cells. The colony number per field was calculated and is shown in the right panel; n=3, *P < 0.05, **P< 0.01, *** P< 0.001. (**I**) Representative images of the migratory cells by Transwell assay were captured 24 h after the cells were inoculated, and the results are summarized in the right panel; n=3, *P < 0.05, **P< 0.01, *** P< 0.001. (**J**) Representative images of the HepG2 cell wound-healing assay were captured at 0, 24, 48 and 72 h after scratching. The wound closure distance was measured with the software from the Leica Application Suite; n=3, *P < 0.05, **P < 0.01 and ***P < 0.001. (**K**) RT-PCR assay of EMT markers was performed in HepG2 cells infected with PLKO.1, sh-*miat*, or coinfected sh-*miat* with the *miR-22-3p* (miR-22 OE)/*miR-22-3p* inhibitor (miR-22 KD). The bars represent the mean and SD of three independent experiments, *P < 0.05, **P< 0.01, *** P< 0.001.

Subsequently, we performed rescue assays to verify the involvement of *miR-22-3p* in the *miat*-mediated effects on cellular senescence and HCC tumorigenesis. The results showed that *miR-22-3p* knockdown rescued and *miR-22-3p* overexpression enhanced the senescence-like phenotypes induced by sh- *miat* in 2BS cell lines (Figure 4A–4D). Similar effect was found in two another human fibroblast cell lines, IMR90 (Supplementary Figure 5A–5D) and MRC-5 (Supplementary Figure 5E–5H). Moreover, *miR-22-3p* overexpression enhanced sh- *miat* induced inhibition of cell proliferation (Figure 4F), cell cycle alteration (Figure 4G), colony formation (Figure 4H), migration (Figure 4I, 4J) and EMT transformation (Figure 4K) in HepG2 and SMMC-7721 cells (Supplementary Figure 5I–5O). However, *miR-22-3p* KD rescued the malignant phenotypes of HCC cells induced by sh-*miat* (Figure 4E–4K and Supplementary Figure 5I–5O). In summary, we found that *miR-22-3p* is essential for sustaining senescence-like phenotypes and tumor-inhibiting effect induced by *miat* downregulation.

### *Sirt1* is a direct target of *miR-22-3p*

**Figure 5:**
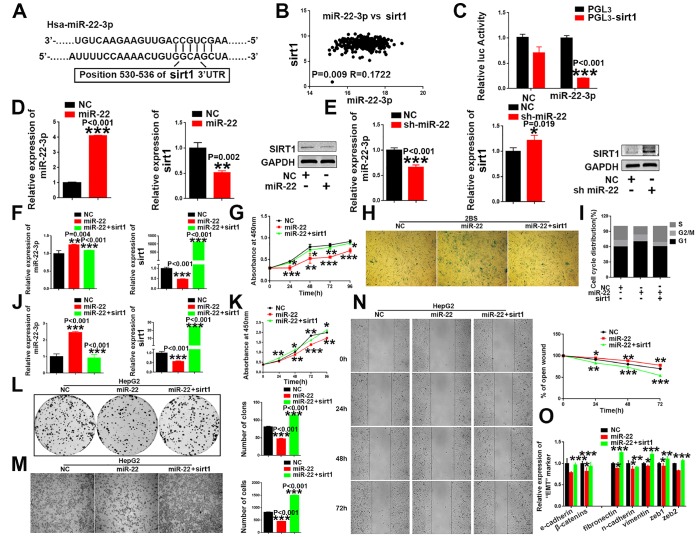
***sirt1* is a downstream target of *miR-22-3p*.** (**A**) Schematic representation of *miR-22-3p* predicted binding site in the 3′-UTR of *sirt1* mRNAs. (**B**) Spearman's correlation coefficient analysis of *miat* and *miR-22-3p* expression in TCGA database. (**C**) *Sirt1* cDNA was cloned downstream of the luciferase gene (PGL3-*sirt1*) and transfected into HepG2 cells with *miR-22-3p* or NC. Luciferase activity was detected 48 h after transfection. The bars represent the mean and SD of three independent experiments, *P < 0.05, **P< 0.01, *** P< 0.001. (**D**) The mRNA and protein levels of *sirt1* were decreased when HepG2 cells were transfected with *miR-22-3p* (miR-22). The bars represent the mean and SD of three independent experiments, *P < 0.05, **P< 0.01, *** P< 0.001. (**E**) The mRNA and protein levels of *sirt1* were increased when HepG2 cells were transfected with the *miR-22-3p* inhibitor (sh-miR-22). Data are expressed as the mean ± SD. n=3. *P < 0.05, **P < 0.01 and ***P < 0.001compared with the control group. (**F**) The mRNA levels of *miR-22-3p* and *sirt1* in 2BS cells infected with the NC, *miR-22-3p* or co-infected with the *miR-22-3p* with *sirt1*. The bars represent the mean and SD of three independent experiments, *P < 0.05, **P< 0.01, *** P< 0.001. (**G**) Cell proliferation was measured using CCK-8 assays in 2BS cells infected with the NC, *miR-22-3p* or co-infected with the *miR-22-3p* with *sirt1*. Data are expressed as the mean ± SD. n=4. *P < 0.05, **P < 0.01 and ***P < 0.001compared with the control group. (**H**) Cellular senescence assay by SA-β-gal staining in 2BS cells infected with the NC, *miR-22-3p* or co-infected with the *miR-22-3p* with *sirt1*. (**I**) Cell cycle assays were performed in 2BS cells infected with the NC, *miR-22-3p* or co-infected with the *miR-22-3p* with *sirt1*. (**J**) The mRNA levels of *miR-22-3p* and *sirt1* in HepG2 infected with the NC, *miR-22-3p* or co-infected with the *miR-22-3p* with *sirt1*. The bars represent the mean and SD of three independent experiments, *P < 0.05, **P< 0.01, *** P< 0.001. (**K**) Cell proliferation was measured using CCK-8 assays in HepG2 cells infected with the NC, *miR-22-3p* or co-infected with the *miR-22-3p* with *sirt1*. The bars represent the mean and SD of three independent experiments, *P < 0.05, **P< 0.01, *** P< 0.001. (**L**) Cell colony formation assay was performed 14 days after HepG2 cells were infected with the NC, *miR-22-3p* or co-infected with the *miR-22-3p* with *sirt1*. The bars represent the mean and SD of three independent experiments; *P < 0.05, **P< 0.01, *** P< 0.001. (**M**) Transwell assays were captured 24 h after the cells were inoculated, and the results are summarized in the right panel. The bars represent the mean and SD of three independent experiments; *P < 0.05, **P< 0.01, *** P< 0.001. (**N**) Representative images of the HepG2 cell wound-healing assay were captured at 0, 24, 48 and 72 h after scratching. The wound closure distance was measured with the software from the Leica Application Suite. The bars represent the mean and SD of three independent experiments; *P < 0.05, **P < 0.01 and ***P < 0.001. (**O**) RT-PCR assay of EMT markers was performed in HepG2 cells infected with the NC, *miR-22-3p* or co-infected with the *miR-22-3p* with *sirt1*; *P < 0.05, **P < 0.01 and ***P < 0.001.

To further investigate the mechanism by which *miat* acts as a microRNA sponge, the potential target genes of *miR-22-3p* were analyzed by online prediction software (TargetScan v 7.2). Then, we focused on *sirt1* (Figure 5A), which is associated with the HCC tumorigenesis and cellular senescence. Significant negative correlation was observed between SIRT1 expression and *miR-22-3p* expression in HCC tissues (R = 0.1722, P = 0.009) (Figure 5B). Then, we performed a reporter system to verify the above conclusions, and found that the relative luciferase activity was reduced with *miR-22-3p* overexpression (Figure 5C). In addition, we also detected the expression of *miR-22-3p* and *sirt1* in HepG2 cells after transfection with a *miR-22-3p* mimic(miR-22) or *miR-22-3p* inhibitor (sh-miR-22). We found that *sirt1* expression was significantly decreased after *miR-22-3p* OE (Figure 5D), and *sirt1* expression increased compared with that in the control group when *miR-22-3p* inhibited in HepG2 cell (Figure 5E). In addition, the levels of *miR-22-3p* and *sirt1* were evaluated in HCC cellular senescence models induced by oxidative stress (H_2_O_2_) and DNA damage (DOX). We found that the expression of *miR-22-3p* significantly increased in a dose-dependent manner in both senescent models, while *sirt1* expression was significantly decreased in both senescent models (Supplementary Figure 1E, 1F and 1I, 1J). Similar effects were observed in SMMC-7721 (Supplementary Figure 1G, 1H and 1K, 1L).

### Restoration of *sirt1* expression reversed the pro-senescent and tumor-suppressing effect induced by *miR-22-3p* in HCC cells

Then, we validated the involvement of *sirt1* in the *miR-22-3p*-mediated effects cellular senescence and HCC progression, and performed rescue assays. Our study showed that the restoration of *sirt1* attenuates the senescence-like phenotypes induced by *miR-22-3p* in human fibroblast 2BS cells (Figure 5F–5I), MRC-5 (Supplementary Figure 6A–6C) and IMR-90 (Supplementary Figure 6D–6F). In addition, *sirt1* overexpression rescued *miR-22-3p* (Figure 5J) induced inhibition of cell proliferation (Figure 5K), colony formation (Figure 5L), migration (Figure 5M, 5N) and EMT transition (Figure 5O) in HepG2 cells, while *sirt1* knockdown restored the pro-tumorigenic effects of HCC inhibited by miR-22 inhibitor (sh-miR-22) (Supplementary Figure 6G–6P). Similar effects were observed in SMMC-7721, another HCC cell line (Supplementary Figure 7). In general, these results indicated that *sirt1* plays a critical role in anti-senescence and pro-tumorigenic effects in HCC.

### *Miat* upregulates the expression of *sirt1* via sponging *miR- 22-3p*

**Figure 6:**
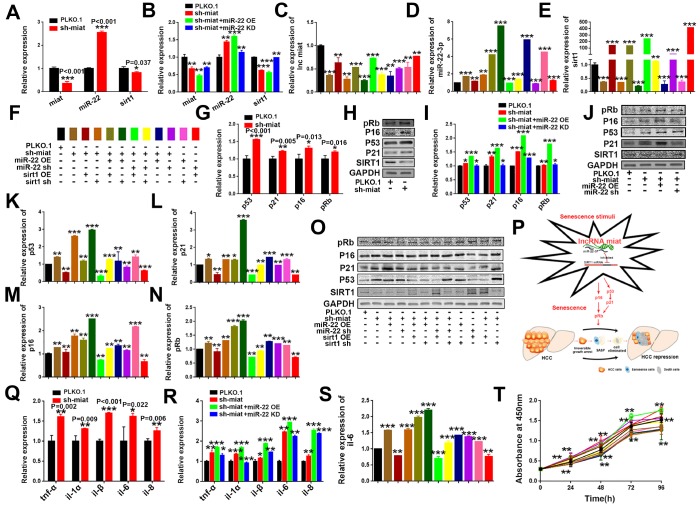
**LncRNA *miat* silencing activates the *p53*/*p21* and *p16/pRb* signaling pathways and promotes the production of SASP in HepG2 cells.** (**A**) RT-PCR analysis for the expression levels of *miat*, *miR-22-3p* and *sirt1* in sh-*miat* HepG2 cells. The bars represent the mean and SD of three independent experiments; *P < 0.05, **P < 0.01 and ***P < 0.001. (**B**) RT-PCR analysis for the expression levels of *miat*, *miR-22-3p* and *sirt1* in HepG2 cells infected with PLKO.1, sh-*miat*, or co-infected with sh-*miat* with the *miR-22-3p* (miR-22 OE) /*miR-22-3p* inhibitor (miR-22 KD). The bars represent the mean and SD of three independent experiments; *P < 0.05, **P < 0.01 and ***P < 0.001. (**C**–**E**) RT-PCR analysis for the expression levels of *miat*, *miR-22-3p* and *sirt1* in HepG2 cells with different treatments. The bars represent the mean and SD of three independent experiments; *P < 0.05, **P < 0.01 and ***P < 0.001. (**F**) The specific color corresponding to different experimental groups in Figure 6C–6E, 6K–6N, 6S, 6T. (**G**) RT-PCR analysis of the expression levels of the *p53*/*p21* and *p16*/*pRb* signaling pathways in sh-*miat* HepG2 cells. The bars represent the mean and SD of three independent experiments; *P < 0.05, **P < 0.01 and ***P < 0.001. (**H**) The protein levels of *sirt1*, *p53*/*p21* and *p16*/pRb were measured by western blotting. (**I**) RT-PCR analysis for the expression levels of the *p53*/*p21* and *p16*/*pRb* signaling pathway in HepG2 cells infected with PLKO.1, sh-*miat*, or co-infected with sh-*miat* with the *miR-22-3p* (miR-22 OE) /*miR-22-3p* inhibitor (miR-22 KD). The bars represent the mean and SD of three independent experiments; *P < 0.05, **P < 0.01 and ***P < 0.001. (**J**) Protein levels of *sirt1, p53*/*p21* and *p16*/*pRb* were measured by western blotting in HepG2 cells infected with PLKO.1, sh-*miat*, or co-infected with sh-*miat* with the *miR-22-3p* (miR-22 OE) /*miR-22-3p* inhibitor (miR-22 KD). (**K**–**N**) RT-PCR analysis for the expression levels of the *p53*/*p21* and *p16*/*pRb* signaling pathway in HepG2 cells with different treatments as described in Figure 6F. The bars represent the mean and SD of three independent experiments; *P < 0.05, **P < 0.01 and ***P < 0.001. (**O**) Protein levels of *sirt1*, *p53*/*p21* and *p16*/*pRb* were measured by western blotting in HepG2 cells with different treatments. (**P**) Schematic overview of the study design. (**Q**) RT-PCR analysis for the expression of selected SAS P components (*tnf-α*, *il-1α, il-1β, il-6* and *il-8*) was analyzed by quantitative PCR in sh-*miat* HepG2 cells. The bars represent the mean and SD of three independent experiments; *P < 0.05, **P < 0.01 and ***P < 0.001. (**R**) RT-PCR analysis for the expression of selected SASP components (*tnf-α*, *il-1α, il-1β, il-6* and *il-8*) was analyzed by quantitative PCR in HepG2 cells infected with PLKO.1, sh-*miat*, or co-infected with sh-*miat* with the *miR-22-3p* (miR-22 OE) /*miR-22-3p* inhibitor (miR-22 KD). The bars represent the mean and SD of three independent experiments; *P < 0.05, **P < 0.01 and ***P < 0.001. (**S**) RT-PCR analysis for the expression of selected SASP components. *il-6* was analyzed by quantitative PCR in HepG2 cells with different treatments. The bars represent the mean and SD of three independent experiments; *P < 0.05, **P < 0.01 and ***P < 0.001. (**T**) Cell proliferation was measured using CCK-8 assays in HepG2 cells with different treatments; n=4, *P < 0.05, **P < 0.01 and ***P < 0.001.

Increasing evidence suggests that lncRNAs can act as ceRNAs and sequester specific miRNAs away from their target genes, consequently inhibiting miRNA stability and functions [36, 37]. Here, we found that knockdown of *miat* could inhibit the expression of *sirt1* (Figure 6A and Supplementary Figure 8A), whereas these effects were significantly strengthened with *miR-22-3p* overexpression, but reversed after *miR-22-3p* KD in both two HCC cell lines (Figure 6B and Supplementary Figure 8B). To explore the functional connection between *miat*, *miR-22-3p* and *sirt1*, we transfected *miR-22-3p* normal control (NC), mimic or inhibitor, or co-infected with *sirt1* or sh-*sirt1* to sh-*miat* HCC cells as Figure 6F. We found that the effects of sh-*miat* on the regulation of *sirt1* expression were partially attenuated by the *miR-22-3p* or inhibitor in HCC cell lines HepG2 (Figure 6C–6E) and SMMC-7721 (Supplementary Figure 8C–8E). These data indicated that *miat* upregulate the expression of *sirt1* by competitively binding to *miR-22-3p*, and *miat* released *sirt1* from *miR-22-3p*.

### Knockdown of *miat* promoted the progression of senescence and activated the tumor suppressor pathway*p53*/*p21* and*p16*/*pRb*

As we known, hallmarks of cellular senescence include telomere erosion, growth inhibition, cell cycle arrest, DNA damage, oxidative stress, tumor suppressor proteins activation (such as *p53, p21, p16 and pRb*) and senescence-associated secretory phenotype (SASP) [15, 38, 39]. We also explore the potential effect of *miat* on *p53*/*p21* and *p16/pRb* signalling pathway in HCC cellular senescence induced by sh-*miat*. The results showed that *miat* knockdown significantly enhanced the mRNA and protein expression of *p53*, *p21, p16* and *pRb* (Figure 6G, 6H and Supplementary Figure 8F, 8G), and these effects were significantly strengthened with *miR-22-3p* overexpression, but reversed after inhibition of *miR-22-3p* in HCC cell lines (Figure 6I, 6J and Supplementary Figure 8H, 8I). In addition, the effects of sh-*miat* on the regulation of *p53*/*p21* and *p16*/*pRb* signalling pathway activity were partially attenuated by *miR-22-3p* KD or *sirt1* overexpression in both HepG2 (Figure 6K–6O) and SMMC-7721cells (Supplementary Figure 8J–8N). Taken together, our data suggested that downregulation of *miat* broke the balance of *miat/miR-22-3p/sirt1* axis, formed “Senescence stimulation” and induced HCC cellular senescence. which activated tumor suppressor pathway *p53/p21*, *p16/pRb* and SASP secretion, resulting in inhibition of HCC cell proliferation and tumorigenesis (Figure 6P).

### Knockdown of *miat* promoted senescent HCC cells secrete SASP and restricted the proliferation of HCC cells

Studies have shown that senescent cells secrete multiple inflammatory proteins known as the SASP, and tumor cells can be eliminated through cellular senescence and SASP [40]. Thus, we next investigated the change of SASP HCC cellular senescence induced by *miat*. We detected the significant induction of major pro-inflammatory cytokines, such as *tnf-α*, *il-1α*, *il-1β*, *il-6*, and *il-8* in *miat* silenced HepG2 cell lines and found that *miat* silencing promotes the secretion of SASP in HCC cell lines (Figure 6Q and Supplementary Figure 8O). These effects were significantly strengthened with *miR-22-3p* overexpression, but reversed after *miR-22-3p* knockdown in both HCC cell lines (Figure 6R and Supplementary Figure 8P). We also detected the expression of *il-6* in HepG2 and SMMC-7721 cells with different treatments as described in Figure 6F or Supplementary Figure 8S, and obtained a similar result that sh- *miat* /*miR-22-3p*/sh-*sirt1* promoted the secretion of SASP in HCC cell lines, and *miR-22-3p* inhibition or *sirt1* overexpression reversed the SASP generation induced by *miat* silencing (Figure 6S and Supplementary Figure 8Q). Certain SASP components, such as *il-6,il-8, also* obviously act in an autocrine loop to reinforce several aspects of senescence including growth arrest. Finally, we detected the ability of cell proliferation in HCC cell lines and verified that sh-*miat* /*miR-22-3p*/sh-*sirt1* silencing inhibited HCC cell proliferation, and these tumor suppressive effects were significantly reversed after *miR-22-3p* KD or *sirt1* OE in both HCC cell lines (Figure 6T and Supplementary Figure 8R).

In summary, our study demonstrates that SAL-*miat* play an important role in HCC cellular senescence, which might offer a potential therapeutic strategy for HCC treatment.

## DISCUSSION

HCC is the fifth most frequently occurring cancer worldwide with poor prognosis. Although much efforts have been made to improve the treatment and surveillance status of HCC, the outcome of clinical is still limited. Therefore, a deeper understanding of molecular mechanisms involved in the carcinogenesis and cancer progression of HCC is urgently needed.

Many scientists believe that aging has an inhibitory effect on tumors, and therefore proposes to treat tumors by activating tumor cellular senescence [6, 41]. Studies have also pointed out that aging may promote tumors in a certain genetic background [42]. In our study, we investigated lncRNAs that were differentially expressed during liver cancer progression and cellular senescence by integrating existing data and focused our research on lncRNA *miat*. We found that, sh-*miat* could induce cellular senescence and delay HCC progression, which indicates that *miat* may play a role as a bridge in cell senescence and HCC progression. In addition, Huang etc. found that knockdown *miat* inhibited proliferation and invasion in both *p53* WT HCC cells SK-HEP-1 and *p53*-MUT cells HLE, which suggested that lncRNA *miat* promotes HCC tumorigenesis in *p53* non-dependent pathway. We also performed pro-tumorigenic effect of the *miat* in *p53*-Mut HCC cell line Huh7 and got consistent results. Based on the conclusion of the previous and the present study, we speculated that *miat* may play the same role in *p53* WT and *p53* deficient HCC cells in different ways, but more details have to be further studied.

Recent studies show that lncRNAs are associated with various diseases, such as Alzheimer's disease, coronary artery disease, prostate cancer, lung cancer and HCC [43–45]. It has become largely accepted that lncRNAs act as ceRNAs to sponge miRNAs and further release the targets of miRNAs from translational inhibition and/or degradation [46]. In our study, we found that lncRNA *miat* acted as a ceRNA to upregulate *sirt1* by sponging *miR-22-3p*, influencing cellular senescence and HCC progression. Overall, these results verified that coding and noncoding RNAs may communicate with each other through the ceRNA language [46]. However, the ceRNA activity of SAL-*miat* may enable it to sponge many miRNAs, which can simultaneously target multiple genes, and more detailed interaction networks have yet to be further studied.

Various oncogenes and tumor suppressors have been shown to be involved in the regulation of senescence-inducing pathways, and senescence induction appears to be an important step in the tumor regression [47]. The *p53*/*p21* and *p16*/*pRb* tumor suppressor pathways are the most important regulators of cellular senescence and tumor progression [48]. Study showed that the two tumor suppressor pathways were putative targets for various cancer therapies because of their significant effect on senescence [49, 50]. In our study, we found that *miat* silencing activates the *p53*/*p21* and *p16*/*pRb* pathways, and promotes cell cycle arrest and cell proliferation inhibition, in both normal human fibroblast cell lines and HCC cell lines.

Furthermore, studies have shown that senescent cells secrete cytokines and other factors of SASP, and SASP influences tissue microenvironments and stimulates tumorigenesis [51–55]. However, there are also emerging views reporting that the specific immune can identify and clear senescent hepatocytes [56]. Thus, SASPs appear to be beneficial or deleterious, depending on the biological context. In our study, we found that *miat* silencing induced HCC cellular senescence, which promotes the production of SASP. Perhaps the anti-cancer function of SASP accelerates the clearance of HCC tumor cells to limit HCC progression, or SASP and cell senescence work together to inhibit the development of HCC.

Given the tumor suppressive potential of senescence, determining how HCC cells escape senescence and devising methods to restore senescence to these cells may be important for developing a new therapeutic option for this malignancy. Therefore, our research started with HCC specific senescence-associated lncRNAs, and focused on SAL-*miat*, which plays a critical role in promoting HCC cellular senescence and inhibiting HCC progression. Mechanistically, we reported that *miat* acted as a ceRNA by sponging *miR-22-3p* to upregulated the expression of *sirt1*. Finally, we identified the *miat* silencing could inhibit HCC tumorigenesis by inducing HCC cellular senescence and activating *p53*/*p21* and*p16*/*pRb* signaling pathways, which promote the production of SASP, and that the anticancer function of SASP accelerates the clearance of HCC tumor cells to limit HCC progression.

## MATERIALS AND METHODS

### Human HCC samples and cell lines

Twenty paired fresh-frozen HCC and para-tumor tissue samples were used in quantitative real-time PCR (qRT-PCR) analysis. HCC and para-tumor tissue samples were surgically resected from HCC patients who underwent hepatectomy at the Peking Union Medical College Hospital (PUMCH, Beijing, China) between 2010 and 2014. All diagnoses were confirmed by pathology. This study was approved by the Ethics Committee of PUMCH, and informed consent was obtained from each patient.

The 293T cell line and HCC cell lines HepG2, SMMC-7721, PLC/PRF/5, Huh7 and SK-hep-1 were obtained from Shanghai Cell Bank, Chinese Academy of Sciences, and cultured as recommended by the supplier. Human diploid fibroblast cells 2BS, IMR90 and MRC-5 were obtained from National Institute of Biological Products, Beijing, China, and cultured as recommended by the supplier. Cells were transiently transfected with plasmids using Lipofectamine 2000 Reagent (Invitrogen), and siRNAs were transfected using Lipofectamine RNAiMAX Reagent (Invitrogen, Carlsbad, USA) following the manufacturer’s protocol. Forty-eight hours after transfection, cells were harvested and lysed to evaluate the transfection efficiency.

### RNA isolation and qRT-PCR) analysis

Total RNA was extracted from cultured cell lines or tumor tissues using TRIzol reagent (Invitrogen, Carlsbad, CA, USA) following the manufacturer’s instructions. RNA was quantified by absorbance at 260 nm. Complementary DNA (cDNA) was then synthesized using the TaKaRa Reverse Transcription System (TaKaRa, Dalian, China). qRT-PCR analysis was performed on the ABI-7500 Real-Time PCR System using iQTM SYBR Green Supermix (Bio-Rad, Hercules, USA) reagent. The relative expression of target genes was calculated using the 2−ΔΔCt method.

### *SA-β-Gal* activity analysis

The activity of *SA-β-gal*, a marker of cellular senescence, was determined by using the Cellular Senescence Assay Kit (Chemicon International, Temecula, CA) according to the manufacturer’s instructions. After 72 h of transfection as indicated above, cells were washed twice with phosphate-buffered saline (PBS), fixed with 1× fixing solution and incubated in 10 cm^2^ flasks at a cell density of 2 × 10^5^ cells/flask at room temperature for 10 min. After removing the fixing solution, cells were washed twice with PBS and incubated overnight with freshly prepared 1× SA-β-gal detection solution at 37°C, without CO_2_ and protected from light. The percentages of blue-stained senescent cells (*SA-β-gal*-positive) were determined by counting 150-200 cells in six microscopic fields.

### Western blot analysis

Cells were collected in RIPA buffer with protease inhibitor cocktails (AMRESCO) and lysed on ice for 30 min with a short vortex every 10 min. Lysates were centrifuged for 15 min at 13,000 × g and 4°C, supernatants were collected, and protein concentrations were determined by BCA Protein Assay Reagent (Pierce). Lysates were fractionated by SDS-PAGE and transferred onto nitrocellulose membranes. For western blotting analysis, the membranes were incubated with primary antibodies against *sirt1*, *p53*, *p21*, *p16* and *pRb* (Abcam, Cambridge, MA, USA) or GAPDH (Santa Cruz Biotechnology, CA, USA) at 4°C overnight. After three washes with TBST, the membranes were incubated with a secondary antibody at room temperature for 2 h. Then, the signals were detected by enhanced chemiluminescence or fluorescence according to the manufacturer’s recommendations.

### Cell proliferation and colony formation assay

Cell viability was measured with the Cell Counting Kit-8 (CCK-8) (Dojindo, Shanghai, China) according to the manufacturer’s instructions. Cells were plated at a density of 1×10^3^ cells per well in 96-well plates and incubated at 37°C. Proliferation rates were determined at 0, 24, 48, 72 and 96 h post transfection, and quantification was performed on a microliter plate reader (Spectra Rainbow, Tecan) using the Clone Select Imager System (Genetix) according to the manufacturer’s protocol. Values represent the mean ± standard deviation (SD) of four data points from a representative experiment, and experiments were repeated more than three times with similar results. Briefly, transfected cells were plated in six-well plates at a density of 1000 cells per well. The medium was changed every 3 days. After 2 weeks, colonies were fixed with methanol and stained with crystal violet for 20 min. Each experiment was repeated at least three times.

### Cell cycle analysis

Cells with different treatments were washed three times with PBS, detached with 0.25% trypsin, and fixed with 75% ethanol at 4°C overnight. After treatment with 2.5 μl of 10 mg/ml RNase A (Fermentas) at 37°C for 30 min, the cells were resuspended in 300 μl of PBS and stained with propidium iodide in the dark for 30 min. The cells were filtered, and fluorescence was measured

### Wound-healing migration assay

To perform migration assays, we seeded cells in confluent monolayers in six-well plates after transfection. A linear gap was generated by scratching the cell layer at the bottoms of the wells using a sterile 200 μl pipette tip. Phase contrast images were acquired at an identical location at 0, 24, 48, and 72 h after scratching, and the width (W) of the scratch wound was measured. The rate of closure of the open wounds was calculated. All measurements were performed in triplicate at least three times.

### Transwell migration assay

Double-chamber migration assays were performed using Transwell chambers (24-well plate, 8 mm pores; BD Biosciences). In brief, the lower chambers were filled with 600 μl of DMEM containing 10% fetal bovine serum (FBS). HCC cells with different treatments were suspended in serum-free medium, seeded in the upper chambers and incubated at 37°C for 24 h. Then, the cells on the upper surface of the filters were removed using cotton wool swabs. The migrated cells on the lower side of the membrane were fixed in 95% methanol and stained with 0.1% crystal violet dye, and the number of cells migrating to the lower surface was counted in three randomly selected high-magnification fields (100×) for each sample.

### Luciferase reporter assay

For reporter gene assays, the constructed luciferase reporter vectors and Renilla vectors as loading controls were cotransfected using Lipofectamine 2000 (Invitrogen) following the manufacturer’s instructions. Forty-eight hours later, cell lysates were collected, and luciferase activity was measured using the Dual-Luciferase Reporter Assay System (Promega) and normalized to Renilla luciferase activity.

### Subcutaneous tumor model

All animal procedures were performed according to the National Animal Experimentation Guidelines upon approval of the experimental protocol by the Institutional Animal Experimentation Committee of PUMCH. For subcutaneous xenograft experiments, BALB/c mice (female, 6-8 weeks of age) were used to examine tumorigenicity. The SMMC-7721 cell line (3 × 10^6^ cells/mouse) with stable knockdown *miat* and the corresponding controls were subcutaneously injected into the nude mice. The size of the tumors was measured by calipers twice a week, and tumor volumes were calculated using the following formula: 1/2 × d^2^ × D. The mice were sacrificed after 4–6 weeks, and tumors were removed for assessment.

### Target prediction

The interactions of lncRNA *miat* and miRNA hsa-*miR-22-3p* were predicted by the online bioinformatics algorithms : starBase v2.0 (http://starbase.sysu.edu.cn/index.php). The target genes of has-*miR-22-3p* were predicted by the online bioinformatics algorithms: TargetScan Release 3.1 (http://www.targetscanmamm31/).

### Transcriptome sequencing and bioinformatics analysis

The gene expression of HCC patients from The Cancer Genome Atlas (TCGA) Liver Cancer (LIHC) dataset was obtained by using the UCSC Xena Browser (https://xenabrowser.net/). The gene expression of proliferating and senescent WI-38 human diploid fibroblasts from the GSE77675 dataset was downloaded from the Gene Expression Omnibus (GEO) database (https://www.ncbi.nlm.nih.gov/gds). SA-LncRNAs and differentially expressed (DE) HCC lncRNAs were determined by t-tests (FDR < 0.1; fold change ≥2 or ≤0.5) in GSE77675 and TCGA datasets. HCC-specific SALncRNAs were then identified by intersecting SALncRNAs and HCC-DElncRNAs. The following lncRNAs were finally reserved for scientific consideration: (1) lncRNAs that were upregulated in both senescent WI38 cells and HCC tumor tissues; and (2) lncRNAs that were downregulated in both senescent WI38 cells and HCC tumor tissues.

### Statistical analysis

All statistical analyses were performed using GraphPad Prism Software and R software (version 3.3.3). For comparisons, Student’s t-test (two-sided), Pearson’s chi-square test, Kaplan–Meier survival analysis, Fisher’s exact test, and Pearson’s correlation analysis were performed as indicated. Each assay was performed in three independent replicates, and all data are expressed as mean ± SD of n = 3 independent samples. A probability of P < 0.05 was considered statistically significant from control. *P < 0.05, **P < 0.01, ***P < 0.001 and ****P < 0.0001.

## Supplementary Material

Supplementary Figures
