# Supplementary material for: lncRNA miat functions as a ceRNA to upregulate sirt1 by sponging miR-22-3p in HCC cellular senescence
Source: Aging (Albany NY). 2019 Sep 10;11(17):7098–122. doi: 10.18632/aging.102240 (PMC6756895; doi:10.18632/aging.102240)
Supplement: Supplementary Figures [file aging-11-102240-s001.pdf]

SUPPLEMENTARY MATERIALS

Supplementary Figures

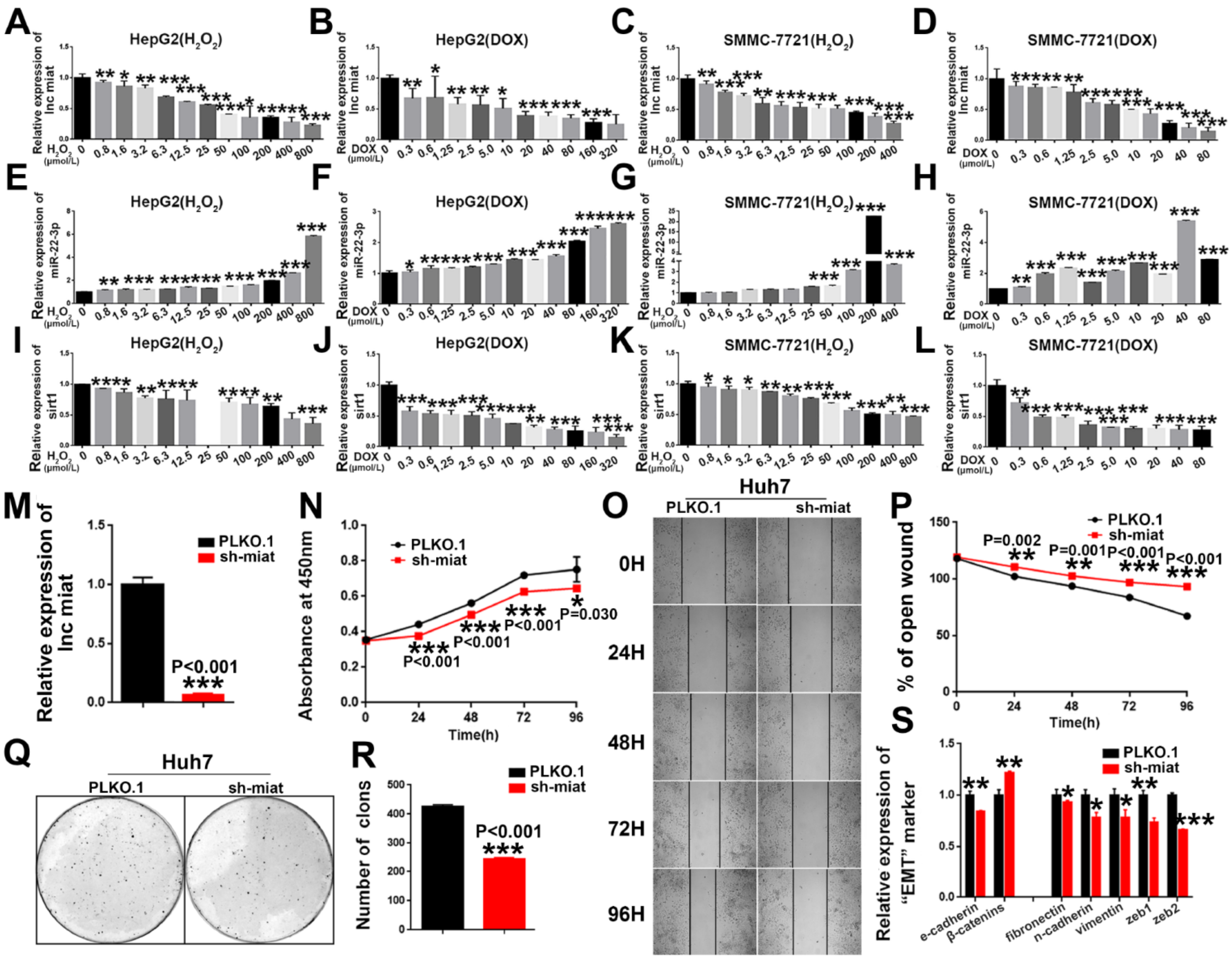

Supplementary Figure 1. The expression of *miat/miR-22-3p/sirt1* in HCC senescence models and knockdown of *miat* suppresses HCC cell hepato-carcinogenesis. (**A**, **B**) The mRNA levels of *miat* in different concentration of H_2O_2 or DOX induced HepG2 cellular senescence models. (**C**, **D**) The mRNA levels of *miat* in different concentration of H_2O_2 or DOX induced SMMC-7721 cellular senescence models. (**E**, **F**) The mRNA levels of *miR-22-3p* in different concentration of H_2O_2 or DOX induced HepG2 cellular senescence models. (**G**, **H**) The mRNA levels of *miR-22-3p* in different concentration of H_2O_2 or DOX induced SMMC-7721 cellular senescence models. (**I**, **J**) The mRNA and protein levels of *sirt1* in different concentration of H_2O_2 or DOX induced HepG2 cellular senescence models. (**K**, **L**) The mRNA and protein levels of *sirt1* in different concentration of H_2O_2 or DOX induced SMMC-7721 cellular senescence models. The bars represent the mean and SD of three independent experiments; *P < 0.05, **P < 0.01 and ***P < 0.001. (**M**) The mRNA levels of *miat* in *miat*-Silenced Huh7 cells. The bars represent the mean and SD of three independent experiments; *P < 0.05, **P < 0.01 and ***P < 0.001. (**N**) Cell proliferation was measured using CCK-8 assays in Huh7 cells with a stable knockdown of *miat,* n=4, *P < 0.05, **P < 0.01 and ***P < 0.001. (**O**, **P**) The wound-healing assay demonstrated that *miat* silencing reduced the migration of Huh7 cells. Representative images were captured at 0 h, 24 h, 48 h and 72 h after scratching. The wound closure distance was measured with the software from the Leica Application Suite. The bars represent the mean and SD of three independent experiments; *P < 0.05, **P < 0.01 and ***P < 0.001. (**Q**, **R**) Cell colony formation assay was performed 14 days after stably knocking down *miat* in Huh7 cells, and the colony number per field was calculated (right). The bars represent the mean and SD of three independent experiments, **P< 0.001, *** P< 0.0001. (**S**) Real-time PCR analysis of the mRNA levels of key EMT markers was performed in Huh7 cells with a stable knockdown of *miat*. The bars represent the mean and SD of three independent experiments; *P < 0.05, **P < 0.01 and ***P < 0.001.

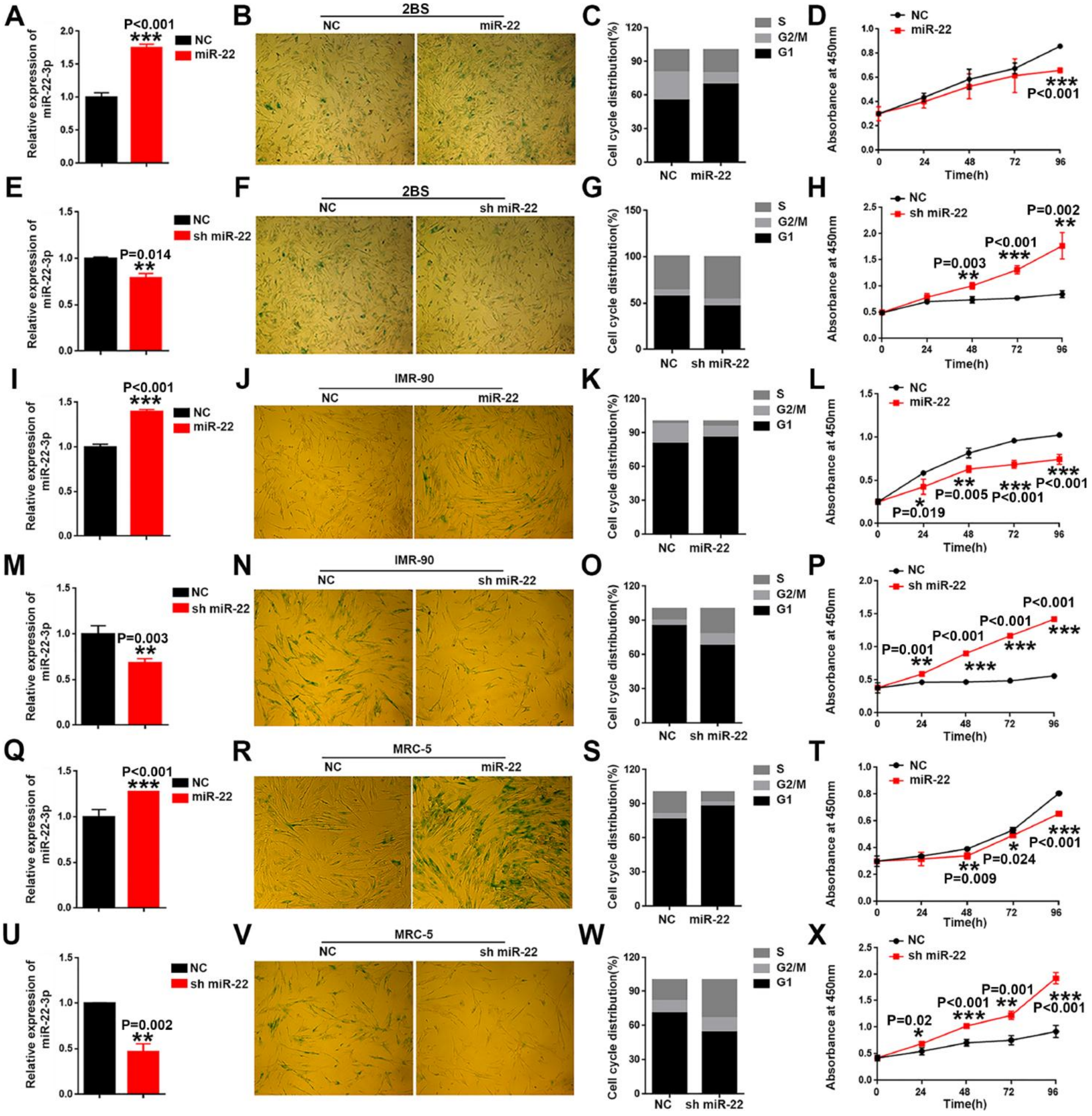

Supplementary Figure 2. MiR-22-3p played an important role in cellular senescence. (**A**, **I**, **Q**) The mRNA levels of *miR-22-3p* in human fibroblast 2BS, IMR-90 and MRC-5 infected with NC or *miR-22-3p* (miR-22). The bars represent the mean and SD of three independent experiments; *P < 0.05, **P < 0.01 and ***P < 0.001. (**B**, **J**, **R**) Cell senescence assay by *SA-β-gal* staining in human fibroblast 2BS, IMR-90 and MRC-5 infected with NC or *miR-22-3p* (miR-22). (**C**, **K**, **S**) Cell cycle assay was performed in human fibroblast 2BS, IMR-90 and MRC-5 infected with NC or *miR-22-3p*(miR-22). (**D**, **L**, **T**) Cell proliferations were measured using CCK-8 assays in normal human fibroblast 2BS, IMR-90 and MRC-5 infected with NC or *miR-22-3p*(miR-22), n=4, *P < 0.05, **P < 0.01 and ***P < 0.001. (**E**, **M**, **U**) The mRNA levels of *miR-22-3p* in normal human fibroblast 2BS, IMR-90 and MRC-5 infected with NC or *miR-22-3p* inhibitor (sh-miR-22). The bars represent the mean and SD of three independent experiments; *P < 0.05, **P < 0.01 and ***P < 0.001. (**F**, **N**, **V**) Cell senescence assay by *SA-β-gal* staining in normal human fibroblast 2BS, IMR-90 and MRC-5 infected with NC or *miR-22-3p* inhibitor (sh-miR-22). (**G**, **O**, **W**) Cell cycle assay was performed in normal human fibroblast 2BS, IMR-90 and MRC-5 infected with NC or *miR-22-3p* inhibitor (sh-miR-22). (**H**, **P**, **X**) Cell proliferations were measured using CCK-8 assays in normal human fibroblast 2BS, IMR-90 and MRC-5 infected with NC or *miR-22-3p* inhibitor (sh-miR-22), n=4, *P < 0.05, **P < 0.01 and ***P < 0.001.

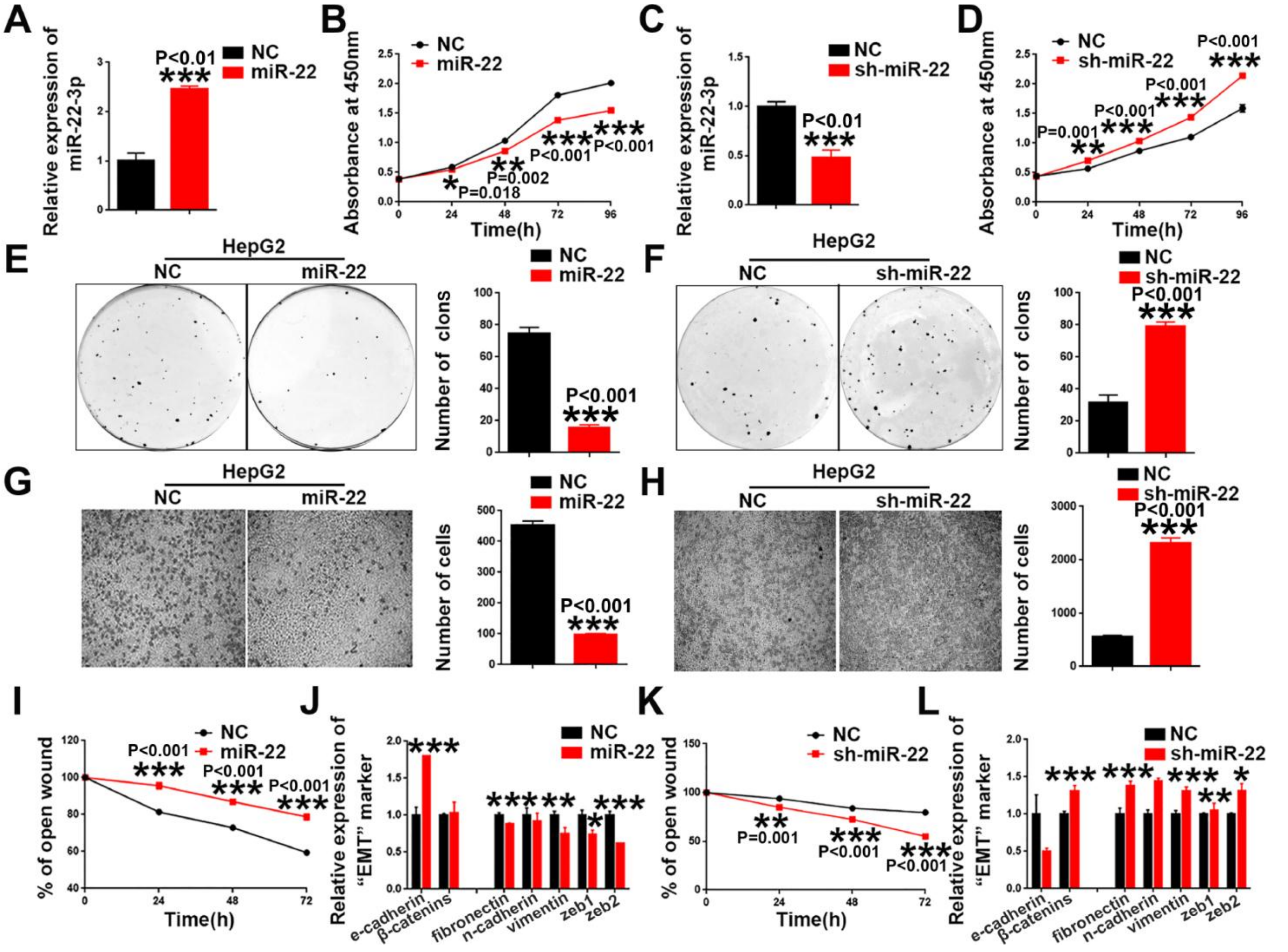

Supplementary Figure 3. MiR-22-3p acts as a tumor suppressor in HCC progression. (**A**, **C**) The mRNA levels of *miR-22-3p* in HepG2 infected with NC or *miR-22-3p*(miR-22) / *miR-22-3p* inhibitor(sh-miR-22). The bars represent the mean and SD of three independent experiments; *P < 0.05, **P < 0.01 and ***P < 0.001. (**B**, **D**) Cell proliferations were measured using CCK-8 assays in HepG2 infected with NC or *miR-22-3p*(miR-22) / *miR-22-3p* inhibitor(sh-miR-22), n=4, *P < 0.05, **P < 0.01 and ***P < 0.001. (**E**, **F**) cell colony formation assay was shown at 14 days after HepG2 infected with NC or *miR-22-3p*(miR-22) / *miR-22-3p* inhibitor(sh-miR-22), and the colony number per field were calculated. The bars represent the mean and SD of three independent experiments. *P < 0.05, **P < 0.01 and ***P < 0.001. (**G**, **H**) Representative images of HepG2 cell transwell migration assay, and the migratory cells were captured 24 hours after the cells were inoculated, respectively, and the results are summarized in the right panel. The bars represent the mean and SD of three independent experiments. *P < 0.05, **P < 0.01 and ***P < 0.001. (**I**, **K**) Representative images of wound-healing assay were captured at 0, 24, 48 and 72h hours after HepG2 infected with NC or *miR-22-3p*(miR-22) / *miR-22-3p* inhibitor (sh-miR-22) (The images were not shown). The wound closure distance was measured with the software from the Leica Application Suite. The bars represent the mean and SD of three independent experiments. *P < 0.05, **P < 0.01 and ***P < 0.001. (**J**, **L**) RT-PCR assay of EMT marker was performed in HepG2cells infected with NC or *miR-22-3p*(miR-22) / *miR-22-3p* inhibitor(sh-miR-22). *p < 0.05, **p < 0.01, ***p < 0.001.

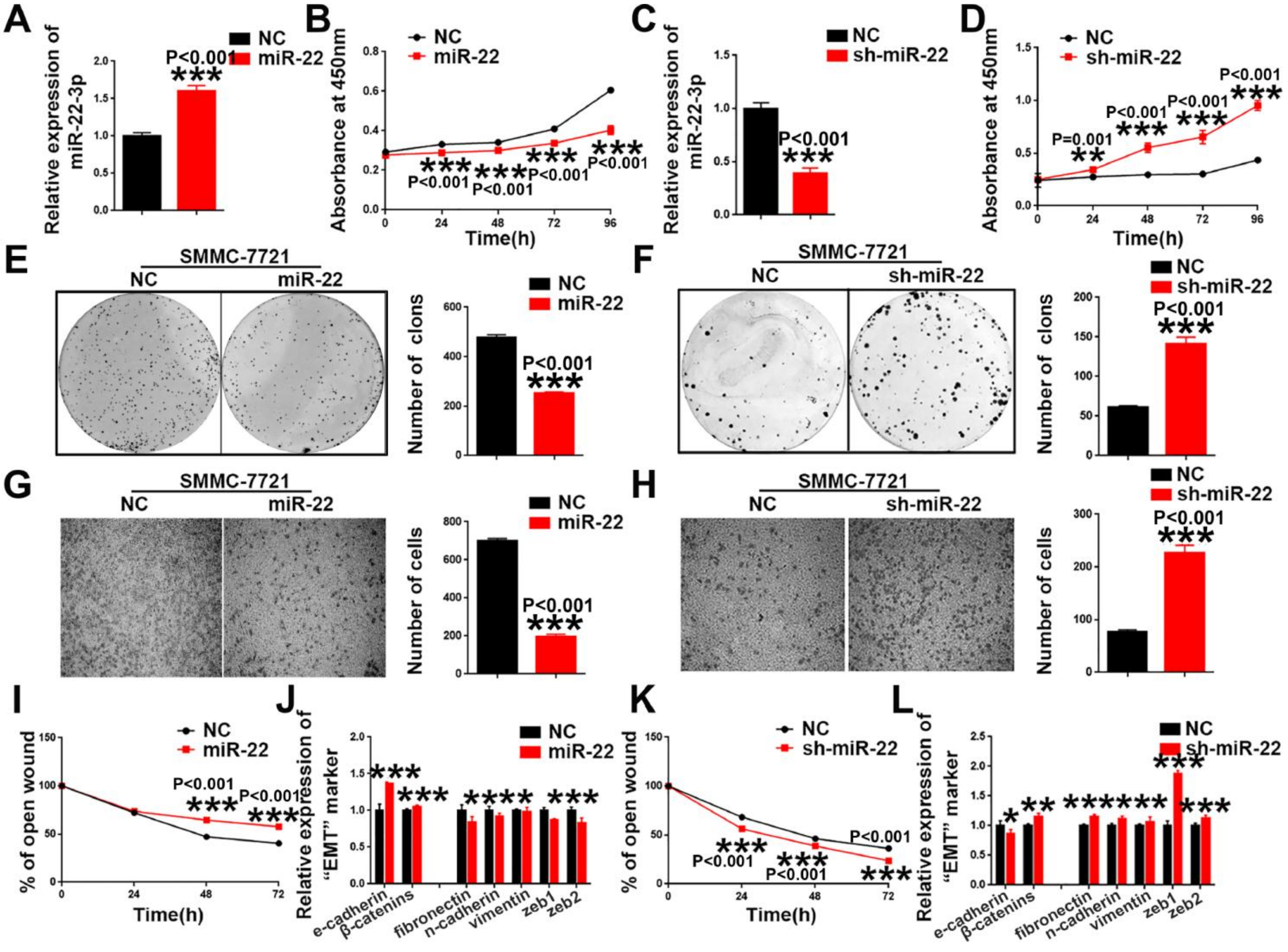

Supplementary Figure 4. MiR-22-3p suppresses SMMC-7721 cell hepato-carcinogenesis. (**A**, **C**) The mRNA levels of *miR-22-3p* in SMMC-7721 infected with NC or *miR-22-3p*(miR-22) / *miR-22-3p* inhibitor(sh-miR-22). The bars represent the mean and SD of three independent experiments; *P < 0.05, **P < 0.01 and ***P < 0.001. (**B**, **D**) Cell proliferations were measured using CCK-8 assays in SMMC-7721 infected with NC or *miR-22-3p*(miR-22) / *miR-22-3p* inhibitor (sh-miR-22), n=4, *P < 0.05, **P < 0.01 and ***P < 0.001. (**E**, **F**) cell colony formation assay was shown at 14 days after SMMC-7721 infected with NC or *miR-22-3p*(miR-22) / *miR-22-3p* inhibitor(sh-miR-22), and the colony number per field were calculated. The bars represent the mean and SD of three independent experiments. **p < 0.01, ***p < 0.001. (**G**, **H**) Representative images of SMMC-7721 cell transwell assay, and the migratory cells were captured 24 hours after the cells were inoculated, respectively, and the results are summarized in the right panel. The bars represent the mean and SD of three independent experiments. ***p < .001. (**I**, **K**) Representative images (The data was not shown) of wound-healing assaywere captured at 0, 24, 48 and 72h hours after SMMC-7721 infected with NC or *miR-22-3p*(miR-22) / *miR-22-3p* inhibitor(sh-miR-22). The wound closure distance was measured with the software from the Leica Application Suite. The bars represent the mean and SD of three independent experiments. *P < 0.05, **P < 0.01 and ***P < 0.001. (**J**, **L**) RT-PCR assay of EMT marker was performed in SMMC-7721cells infected with NC or miR-22 mimic/ miR-22 inhibitor. *p < 0.05, **p < 0.01, ***p < 0.001.

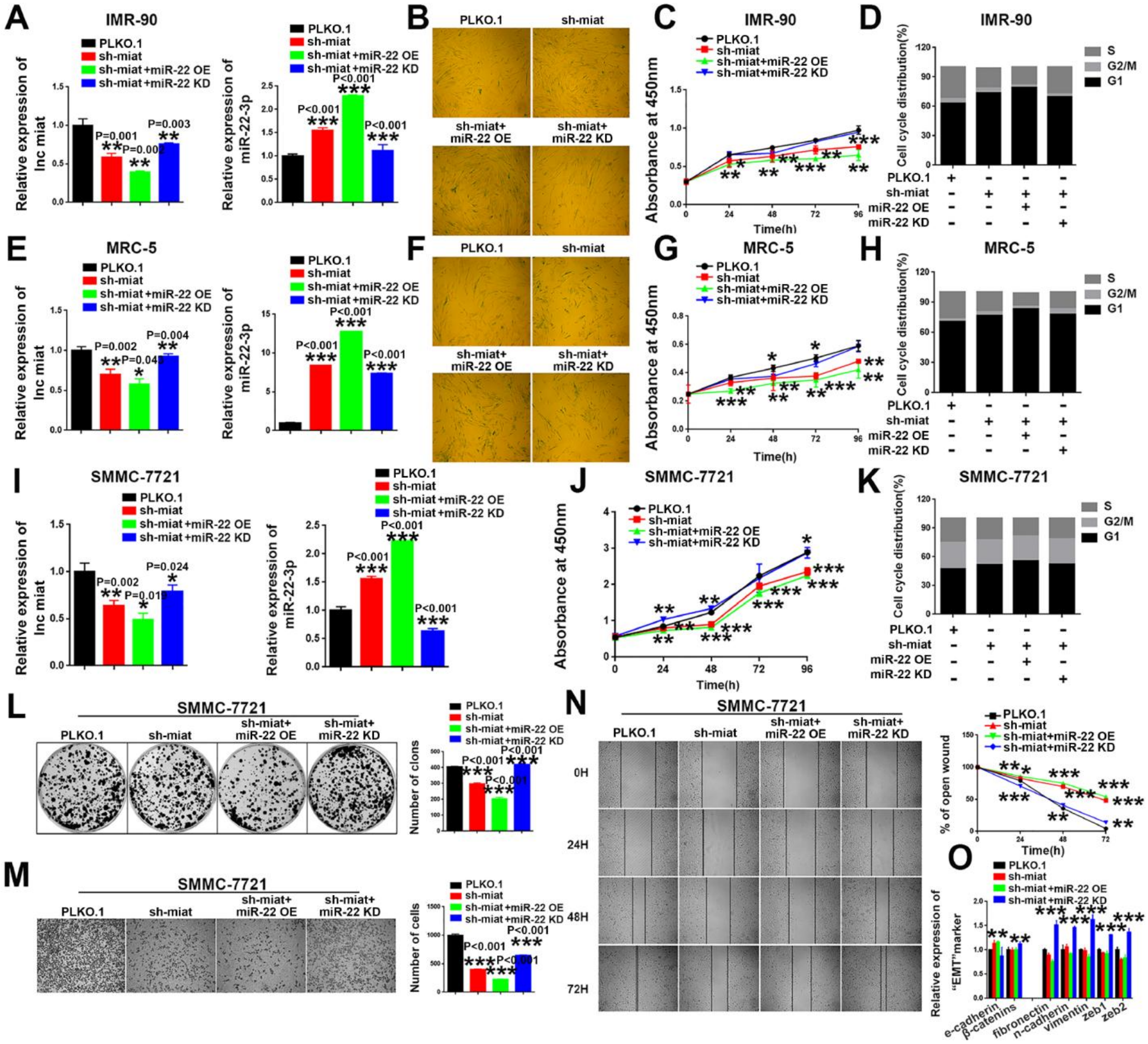

Supplementary Figure 5. *MiR-22-3p* is essential for sustaining senescence-like phenotypes and hepatic inhibition induced by sh-*miat*. (**A**, **E**) The mRNA levels of *miat* and *miR-22-3p* in IMR-90 and MRC-5 cells infected with PLKO.1, sh-*miat*, or coinfected sh-*miat* with the *miR-22-3p* (miR-22 OE)/*miR-22-3p* inhibitor (miR-22 KD). The bars represent the mean and SD of three independent experiments; *P < 0.05, **P < 0.01 and ***P < 0.001. (**B**, **F**) Cellular senescence assay by *SA-β-ga*l staining in IMR-90 and MRC-5 cells infected with PLKO.1, sh-*miat*, or coinfected sh-*miat* with the *miR-22-3p* (miR-22 OE)/*miR-22-3p* inhibitor (miR-22 KD). (**C**, **G**) Cell proliferation was measured using CCK-8 assays in IMR-90 and MRC-5 cells infected with PLKO.1, sh-*miat*, or coinfected sh-*miat* with the *miR-22-3p* (miR-22 OE)/*miR-22-3p* inhibitor (miR-22 KD), n=4, *P < 0.05, **P < 0.01 and ***P < 0.001. (**D**, **H**) Cell cycle assay was performed in IMR-90 and MRC-5 cells infected with PLKO.1, sh-*miat*, or coinfected sh-*miat* with the *miR-22-3p* (miR-22 OE)/*miR-22-3p* inhibitor (miR-22 KD). N=3, *P < 0.05, **P < 0.01 and ***P < 0.001. (**I**) The mRNA levels of *miat* and *miR-22-3p* in SMMC-7721 cells infected with PLKO.1, sh-*miat*, or coinfected sh-*miat* with the *miR-22-3p* (miR-22 OE)/*miR-22-3p* inhibitor (miR-22 KD). The bars represent the mean and SD of three independent experiments; *P < 0.05, **P < 0.01 and ***P < 0.001. (**J**) Cell proliferation was measured using CCK-8 assays in SMMC-7721 cells infected with PLKO.1, sh-*miat*, or coinfected sh-*miat* with the *miR-22-3p* (miR-22 OE)/*miR-22-3p* inhibitor (miR-22 KD), n=4, *P < 0.05, **P < 0.01 and ***P < 0.001. (**K**) Cell cycle assay was performed in SMMC-7721 cells infected with PLKO.1, sh-*miat*, or coinfected sh-*miat* with the *miR-22-3p* (miR-22 OE)/*miR-22-3p* inhibitor (miR-22 KD). (**L**) Cell colony formation assay shown at 14 days after infected with PLKO.1, sh-*miat*, or coinfected sh-*miat* with the *miR-22-3p* (miR-22 OE)/*miR-22-3p* inhibitor (miR-22 KD) in SMMC-7721 cells. The colony number per field was calculated and is shown in the right panel; n=3, *P < 0.05, **P < 0.01 and ***P < 0.001. (**M**) Representative images of the migratory cells by transwell assay were captured 24 h after the cells were inoculated, and the results are summarized in the right panel; n=3, *P < 0.05, **P < 0.01 and ***P < 0.001. (**N**) Representative images of the SMMC-7721 cell wound-healing assay were captured at 0, 24, 48 and 72 h after scratching. The wound closure distance was measured with the software from the Leica Application Suite; n=3, *P < 0.05, **P < 0.01 and ***P < 0.001. (**O**) RT-PCR assay of EMT markers was performed in SMMC-7721 cells infected with PLKO.1, sh-*miat*, or coinfected sh-*miat* with the *miR-22-3p* (miR-22 OE)/*miR-22-3p* inhibitor (miR-22 KD); *P < 0.05, **P < 0.01 and ***P < 0.001.

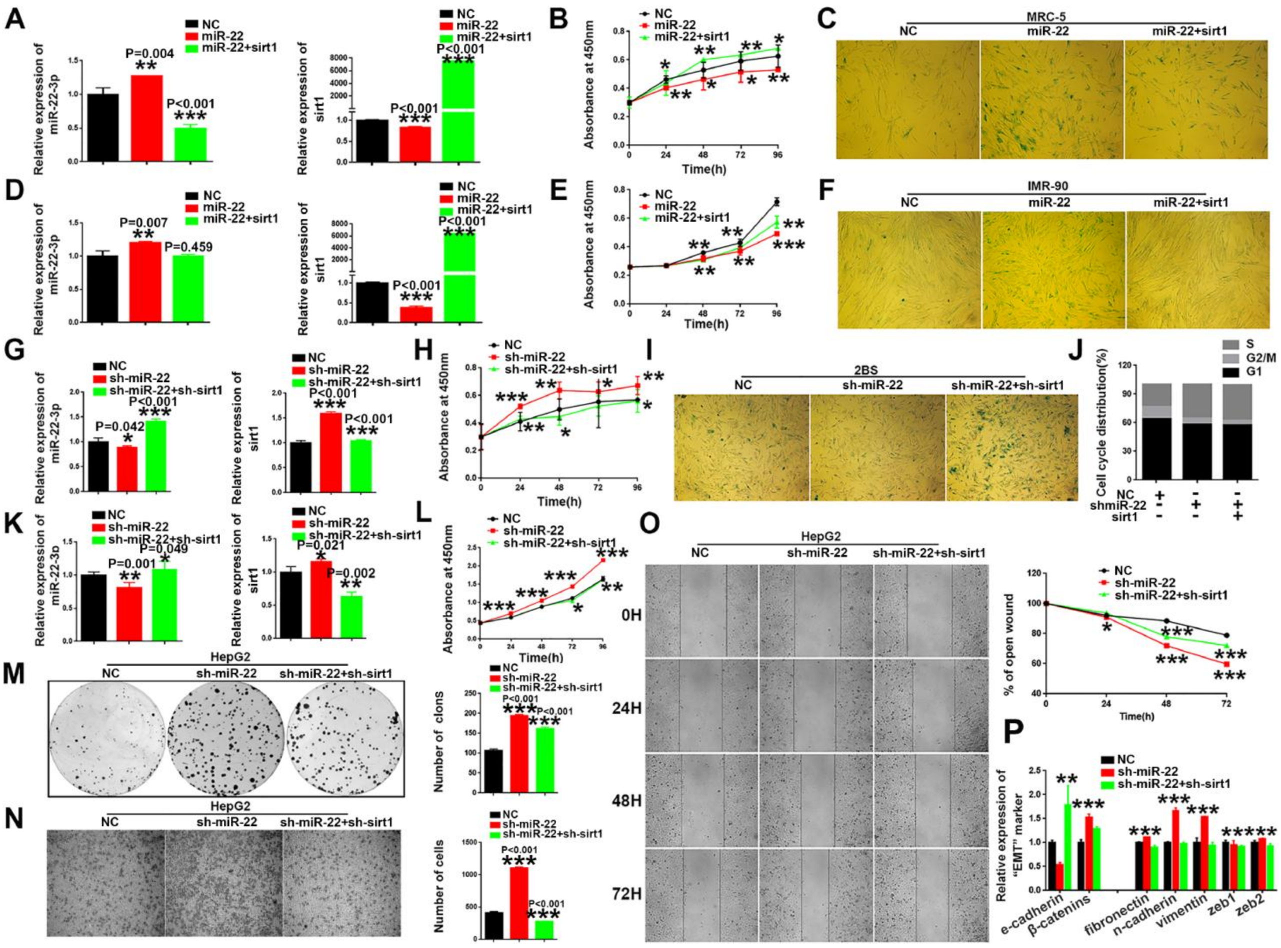

Supplementary Figure 6. *Sirt1* affected *miR-22-3p* mediated cellular senescence and HCC progression. (**A**, **D**) The mRNA levels of *miR-22-3p* and *sirt1* in IMR-90 and MRC-5 infected with NC, *miR-22-3p*(miR-22) or co-infect *miR-22-3p*(miR-22) with *sirt1*. The bars represent the mean and SD of three independent experiments; *P < 0.05, **P < 0.01 and ***P < 0.001. (**B**, **E**) Cell proliferations assay was performed in IMR-90 and MRC-5 infected with NC, *miR-22-3p*(miR-22) or co-infect *miR-22-3p*(miR-22) with *sirt1,* n=4, *P < 0.05, **P < 0.01 and ***P < 0.001. (**C**, **F**) Cell senescence assay by *SA-β-gal* staining in IMR-90 and MRC-5 infected with NC, *miR-22-3p*(miR-22) or co-infect *miR-22-3p*(miR-22) with *sirt1*. (**G**) The mRNA levels of *miR-22-3p* and *sirt1* in 2BS infected with NC, *miR-22-3p* inhibitor (sh miR-22) or co-infect *miR-22-3p* inhibitor (sh miR-22) with sh-*sirt1*. The bars represent the mean and SD of three independent experiments; *P < 0.05, **P < 0.01 and ***P < 0.001. (**H**) Cell proliferations were measured using CCK-8 assays in 2BS cells with different treatment*,* n=4, *P < 0.05, **P < 0.01 and ***P < 0.001. (**I**) Cell senescence assay by *SA-β-gal* staining in 2BS cells with different treatment. (**J**) Cell cycle assay was performed in 2BS cells with different treatment. (**K**) The mRNA levels of *miR-22-3p* and *sirt1* in HepG2 infected with NC, *miR-22-3p* inhibitor (sh miR-22) or co-infect *miR-22-3p* inhibitor (sh miR-22) with sh-*sirt1*. The bars represent the mean and SD of three independent experiments; *P < 0.05, **P < 0.01 and ***P < 0.001. (**L**) Cell proliferations were measured using CCK-8 assays in HepG2 cells with different treatment*,* n=4, *P < 0.05, **P < 0.01 and ***P < 0.001. (**M**) Cell colony formation assay was shown at 14 days after HepG2 cells with different treatment. The bars represent the mean and SD of three independent experiments; *P < 0.05, **P < 0.01 and ***P < 0.001. (**N**) Transwell assay were captured 24 hours after the cells were inoculated, respectively, and the results are summarized in the right panel. The bars represent the mean and SD of three independent experiments; *P < 0.05, **P < 0.01 and ***P < 0.001. (**O**) Representative images of the HepG2 cells wound-healing assay were captured at 0, 24, 48 and 72h hours after scratching. The wound closure distance was measured with the software from the Leica Application Suite. The bars represent the mean and SD of three independent experiments; *P < 0.05, **P < 0.01 and ***P < 0.001. (**P**) RT-PCR assay of EMT marker was performed in HepG2 cells with different treatment. *P < 0.05, **P < 0.01 and ***P < 0.001.

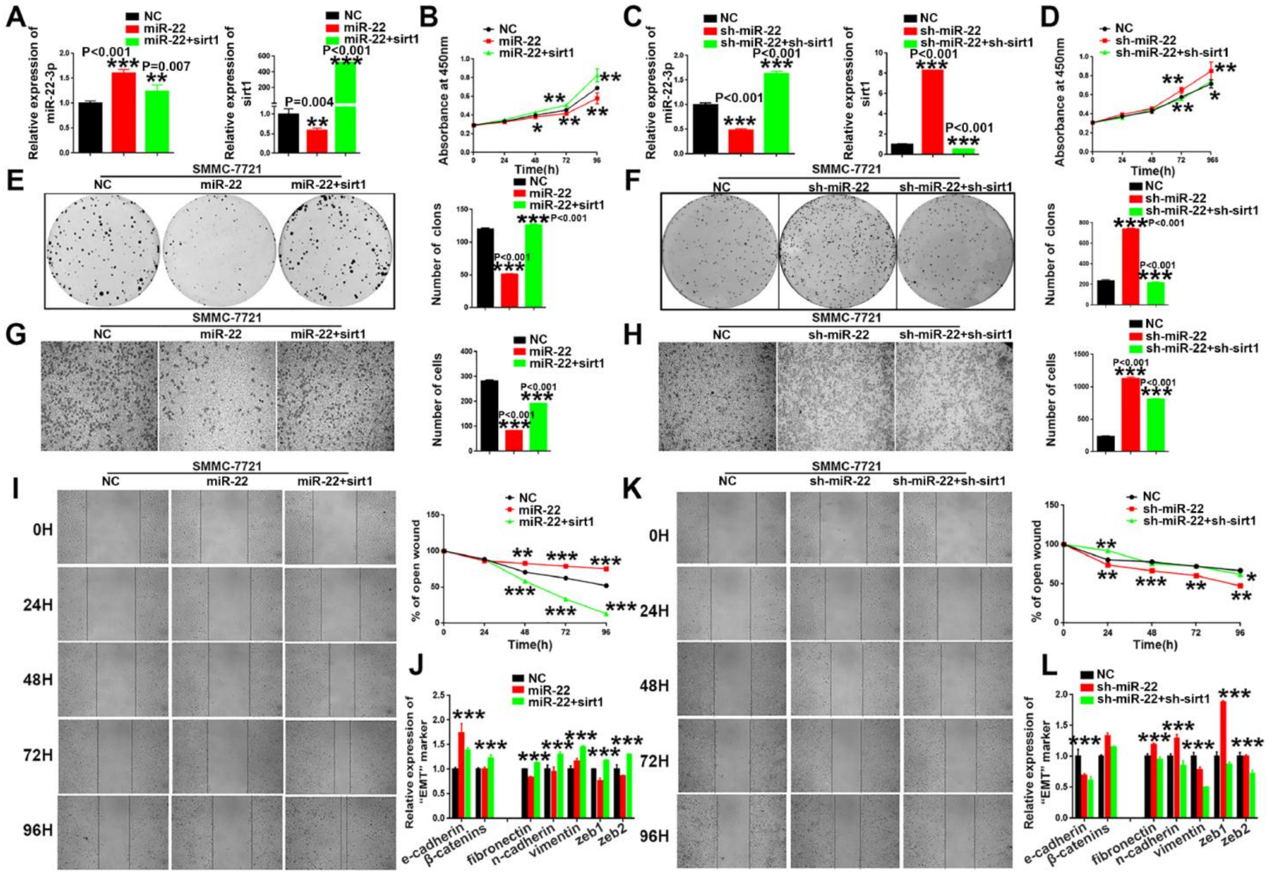

Supplementary Figure 7. *Sirt1* affected *miR-22-3p* mediated HCC progression in SMMC-7721. (**A**, **C**) The mRNA levels of *miR-22-3p* and *sirt1* in SMMC-7721 cells infected with NC, *miR-22-3p* (miR-22) or co-infect *miR-22-3p* (miR-22) with *sirt1*. The bars represent the mean and SD of three independent experiments; *P < 0.05, **P < 0.01 and ***P < 0.001. (**B**, **D**) Cell proliferations were measured using CCK-8 assays in SMMC-7721 cells infected with the NC, *miR-22-3p* (miR-22) or coinfected with the *miR-22-3p* (miR-22) with *sirt1,* n=4, *P < 0.05, **P < 0.01 and ***P < 0.001. (**E**, **F**) Cell colony formation assay was shown at 14 days after SMMC-7721 cells infected with NC, *miR-22-3p* (miR-22) or co-infect *miR-22-3p* (miR-22) with *sirt1*. The bars represent the mean and SD of three independent experiments; *P < 0.05, **P < 0.01 and ***P < 0.001. (**G**, **H**) Transwell assay were captured 24 hours after the cells were inoculated, respectively, and the results are summarized in the right panel. The bars represent the mean and SD of three independent experiments. **P < 0.01 and ***P < 0.001. (**I**, **K**) Representative images of the SMMC-7721 cells wound-healing assay were captured at 0, 24, 48 and 72h hours after scratching. The wound closure distance was measured with the software from the Leica Application Suite. The bars represent the mean and SD of three independent experiments; *P < 0.05, **P < 0.01 and ***P < 0.001. (**J**, **L**) RT-PCR assay of EMT marker was performed in SMMC-7721 cells with different treatment. *P < 0.05, **P < 0.01 and ***P < 0.001.

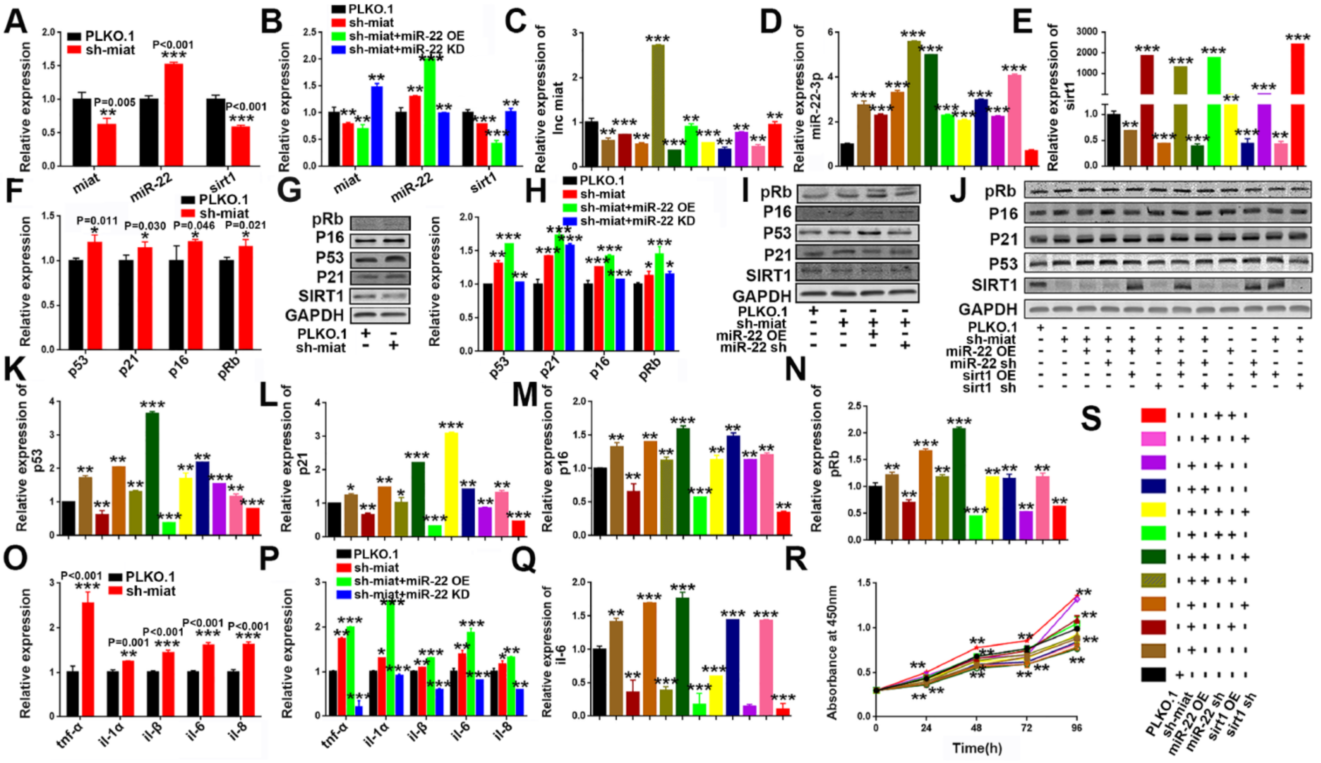

Supplementary Figure 8. Knockdown of *miat* promoted senescent cells secrete SASP and restricted the proliferation in SMMC-7721 cell line. (**A**) RT-PCR analysis for the expression levels of *miat*, *miR-22-3p* and *sirt1* in sh-*miat* SMMC-7721 cells. The bars represent the mean and SD of three independent experiments; *P < 0.05, **P < 0.01 and ***P < 0.001. (**B**) RT-PCR analysis for the expression levels of *miat*, *miR-22-3p* and *sirt1* in SMMC-7721 cells infected with PLKO.1, sh-*miat*, or coinfected with sh-*miat* with the *miR-22-3p* (miR-22 OE) / *miR-22-3p* inhibitor (miR-22 KD). The bars represent the mean and SD of three independent experiments; *P < 0.05, **P < 0.01 and ***P < 0.001. (**C**–**E**) RT-PCR analysis for the expression levels of *miat*, *miR-22-3p* and *sirt1* in SMMC-7721 cells with different treatments. The bars represent the mean and SD of three independent experiments; *P < 0.05, **P < 0.01 and ***P < 0.001. (**F**) RT-PCR analysis of the expression levels of the *p53/p21* and *p16/pRb* signaling pathways in sh-*miat* SMMC-7721 cells. The bars represent the mean and SD of three independent experiments; *P < 0.05, **P < 0.01 and ***P < 0.001. (**G**) The protein levels of *sirt1*, *p53/p21* and *p16/pRb* were measured by western blotting. (**H**) RT-PCR analysis for the expression levels of the *p53/p21* and *p16/pRb* signaling pathway in SMMC-7721 cells infected with PLKO.1, sh-*miat*, or coinfected with sh-*miat* with the *miR-22-3p* (miR-22 OE) / *miR-22-3p* inhibitor (miR-22 KD). The bars represent the mean and SD of three independent experiments; *P < 0.05, **P < 0.01 and ***P < 0.001. (**I**) Protein levels of *sirt1*, *p53/p21* and *p16/pRb* were measured by western blotting in SMMC-7721 cells infected with PLKO.1, sh-*miat*, or coinfected with sh-*miat* with the *miR-22-3p* (miR-22 OE) / *miR-22-3p* inhibitor (miR-22 KD). (**J**) Protein levels of *sirt1, p53/p21* and *p16/pRb* were measured by western blotting in SMMC-7721 cells with different treatments. (**K**–**N**) RT-PCR analysis for the expression levels of the *p53/p21* and *p16/pRb* signaling pathway in SMMC-7721 cells with different treatments. The bars represent the mean and SD of three independent experiments; *P < 0.05, **P < 0.01 and ***P < 0.001. (**O**) RT-PCR analysis for the expression of selected SASP components (*tnf-α, il-1α, il-1β, il-6* and *il-8*) was analyzed by quantitative PCR in sh-*miat* SMMC-7721 cells. The bars represent the mean and SD of three independent experiments; *P < 0.05, **P < 0.01 and ***P < 0.001. (**P**) RT-PCR analysis for the expression of selected SASP components (*tnf-α, il-1α, il-1β, il-6* and *il-8*) was analyzed by quantitative PCR in SMMC-7721 cells infected with PLKO.1, sh-*miat*, or coinfected with sh-*miat* with the *miR-22-3p* (miR-22 OE) / *miR-22-3p* inhibitor (miR-22 KD). The bars represent the mean and SD of three independent experiments; *P < 0.05, **P < 0.01 and ***P < 0.001. (**Q**) RT-PCR analysis for the expression of selected SASP components. *il-6* was analyzed by quantitative PCR in SMMC-7721 cells with different treatments. The bars represent the mean and SD of three independent experiments; *P < 0.05, **P < 0.01 and ***P < 0.001. (**R**) Cell proliferation was measured using CCK-8 assays in SMMC-7721 cells with different treatments; n=4, *P < 0.05, **P < 0.01 and ***P < 0.001. (**S**) The specific color corresponding to different experimental groups in Figure 8C–8E, 8K–8N, 8Q and 8R.
